# Cooperative Catalytic Role of Co and Mn Sites on LaCo_*x*_Mn_1–*x*_O_3_ Perovskite Nanoparticles in CO and NO Oxidation

**DOI:** 10.1021/acsanm.5c02876

**Published:** 2025-08-18

**Authors:** Kerem Emre Ercan, Mustafa Karatok, Zafer Say, Merve Kurt, Abel Tetteh Sika-Nartey, Emrah Ozensoy

**Affiliations:** † Department of Chemistry, 52948 Bilkent University, Ankara 06800, Turkey; § Roketsan Inc., Elmadag, Ankara 06780, Turkey; ‡ Department of Nanotechnology and Nanomedicine, 37515 Hacettepe University, Ankara 06800, Turkey; ¥ Department of Materials Science and Nanotechnology Engineering, TOBB University of Economics and Technology, Ankara 06510, Turkey; ¶ UNAM-National Nanotechnology Center, 52948 Bilkent University, Ankara 06800, Turkey

**Keywords:** perovskite catalysts, CO oxidation, NO oxidation, PGM, oxidation catalysts, oxygen vacancies

## Abstract

Perovskites have significant potential to improve efficiency, reduce the costs of conventional oxidation catalysts, and contribute to cleaner and more sustainable energy solutions. However, numerous structural factors influencing their catalytic performance are still a subject to debate. In this study, simple perovskite nanoparticles in the form of LaCoO_3_ (LC) and LaMnO_3_ (LM), as well as LaCo_*x*_Mn_1–*x*_O_3_ (LCM)-mixed B-site perovskites with different B-site cations, were synthesized and their performances in CO oxidation and NO oxidation reactions were examined. The LaCo_0.8_Mn_0.2_O_3_ catalyst exhibited the highest catalytic activity in both CO and NO oxidation reactions, surpassing the 1 wt %Pt/γ-Al_2_O_3_ benchmark nanoparticle catalyst and other currently investigated perovskite nanoparticles. Co sites (predominantly Co^3+^) in the optimized LaCo_0.8_Mn_0.2_O_3_ catalyst were found to be enriched in electron density, while Mn sites (mostly in Mn^4+^ form) were found to be more electron deficient as opposed to LC and LM. LaCo_0.8_Mn_0.2_O_3_ not only released significantly greater amounts of oxygen and generated larger extents of oxygen vacancies than LC and LM under reducing conditions but also achieved this at favorably lower temperatures. In light of the current results, we report that Co sites in LCM operate as the main active site during both CO and NO oxidation by enabling stabilization and activation of O_2_ (ads), while Mn sites mainly serve as promoters by increasing the adsorption strength of CO (ads) and NO (ads) as well as facilitating oxygen vacancy formation and vacancy regeneration, where oxygen vacancies were also found to contribute particularly to the NO oxidation reaction within the currently investigated thermal window. These findings demonstrate that the electronic properties of LCM can be systematically tailored at the nanometer scale in a versatile manner to address different reactivity requirements of challenging catalytic reactions.

## Graphical abstract

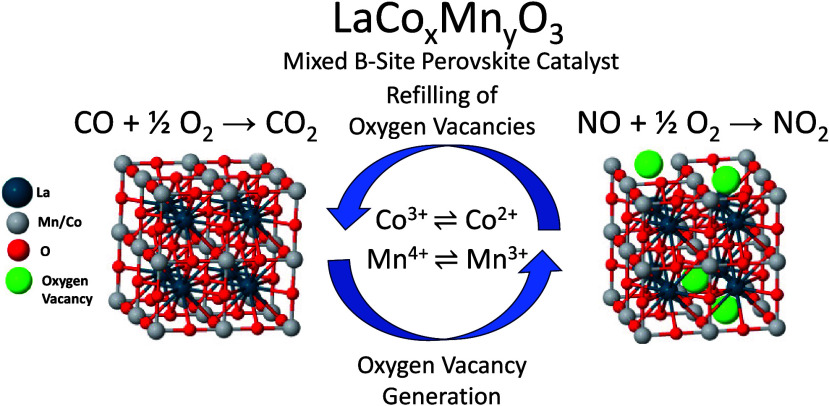

## 1 Introduction

Emissions of air pollutants such as carbon monoxide (CO) and nitrogen oxides (NO_*x*_) due to fossil fuel consumption in mobile systems (*e.g.,* transportation) and stationary applications (*e.g.,* manufacturing and heating) are persistent global issues. Over 400,000 premature deaths related to air pollution occur annually in Europe.^1^ To mitigate these negative impacts, it is essential to develop new catalytic materials that can control gas emissions more efficiently.^2^

Conventional heterogeneous oxidation catalysts used in emission control systems often rely on expensive platinum group metals (PGM) such as Pt, Pd, Rh, Ir, and Ru.^3^ In addition to the efforts focusing on the improvement of the efficiency of existing oxidation catalysts, the design of PGM-free novel catalysts to reduce their costs has been a major scientific and technological challenge for many decades.^4–7^ Perovskites can be considered as promising contenders to achieve these goals. For example, relatively higher CO and NO_*x*_ abatement activity was reported on a Pd-doped perovskite (LaFe_0.57_Co_0.38_Pd_0.05_O_3_) compared to the conventional Pd/Al_2_O_3_ catalyst.^8^ In a later study, higher performance of NO oxidation and a similar activity of NO_*x*_ abatement were reported on PGM-free La_1–*x*_Sr_*x*_CoO_3_ perovskites compared to a benchmark Pt/γ-Al_2_O_3_ catalyst.^9^ High catalytic performance of PGM-free perovskites has also been reported for other classes of reactions.^10–14^ Some of the reasons behind the superior catalytic performance of perovskite materials in specific reactions are yet to be a subject to debate.

Perovskites are materials with an ideal empirical formula of ABO_3_, where the A and B sites host different metal cations. Distinct cation combinations in perovskites offer unique chemical properties, providing a vast library of potential catalysts to be used in a variety of applications. Furthermore, it is known that the redox active *B* ^*x+*^ (*x* = 2, 3, 4) sites in perovskites can readily change their oxidation states under oxidizing/reducing conditions, triggering catalytic action.^9,10,15,16^ However, some of these redox processes are not reversible and thus lead to catalytic aging and loss of activity. Therefore, a precise and comprehensive understanding of the correlation between perovskite B-site electronic structure, oxygen dynamics, and catalytic activity is of immense importance for fine-tuning their catalytic properties for optimum performance.

Changes in the catalytic properties of perovskites due to compositional nonstoichiometries and substitutions have been extensively studied in the literature.^6,17–21^ The presence of cationic and/or anionic defects in perovskites has been reported to enhance redox reversibility of the B-site cations and accelerate the oxide ion or oxygen vacancy mobilities, which may ultimately boost specifically the NO/CO oxidation performances of these systems.^9,15,16,22^ The electronic modification of these simple perovskites that contain a single type of metal on the B site, LaCoO_3_ (LC) and LaMnO_3_ (LM) in particular, leads to an enhancement in either CO or NO oxidation reaction.^9,23^ Here, we hypothesize that simultaneous utilization of Co and Mn cations in the B sites of the perovskite architecture could be a worthwhile synthetic strategy to obtain highly active and durable catalysts for both CO and NO oxidation reactions.

Accordingly, in the current work, we synthesized mixed B-site perovskites in the form of LaCo_*x*_Mn_1–*x*_O_3_ (LCM, where *x* = Co/Mn atomic ratio) containing both Co and Mn B-site cations with various compositions. Electronic and structural properties of the optimized catalyst were studied in detail *via in situ*/*ex-situ* spectroscopy, microscopy, and diffraction techniques in comparison to LaCoO_3_ and LaMnO_3_ simple perovskites (LC and LM). The relationship between the perovskite electronic structure and catalytic performance in the CO and NO oxidation reactions was demonstrated. The LaCo_0.8_Mn_0.2_O_3_ catalyst exhibited the highest activity in both CO oxidation and NO oxidation at lower temperatures compared to the LC and LM, and the 1 wt % Pt/Al_2_O_3_ benchmark catalyst, indicating that tailored perovskites can be promising catalysts in emission control systems, and, by extension, in various other oxidation reactions.^10^

## 2 Methods

### 2.1 Chemicals

La­(NO_3_)_3_·6H_2_O (Sigma-Aldrich, ≥99.5%), Mn­(NO_3_)_2_·4H_2_O (Sigma-Aldrich, ≥97%), Co­(NO_3_)_2_·6H_2_O (Sigma-Aldrich, ≥98%), citric acid (C_6_H_8_O_7_, Sigma-Aldrich, ≥99.5%), Pt­(NH_3_)_2_(NO_2_)_2_ (Sigma-Aldrich, 3.4 wt % solution in dilute NH_3_(aq)), and γ-Al_2_O_3_ powder (Alfa Aesar, 3.2 mm tablets, 175 m^2^/g, ≥96%) were used in the catalyst synthesis without further purification.

### 2.2 Catalyst Preparation

LC and LM catalysts were synthesized using a previously reported method.^24^ This synthesis method was modified to prepare LCM catalysts with two different B-site cations. Note that in the current report, LCM sample names are assigned based on the nominal metal precursor mole ratios (*x* = Co/Mn nominal atomic ratio) initially used in the synthesis, rather than the bulk compositions measured after the synthesis via inductively coupled plasma mass spectroscopy (ICP-MS). Briefly, appropriate amounts of metal precursors (La­(NO_3_)_3_·6H_2_O, Mn­(NO_3_)_2_·4H_2_O, and Co­(NO_3_)_2_·6H_2_O) were added into 0.12 M citric acid solution and stirred for 1 h (see Scheme 1 in the main text and Table S1 in the Supporting Information, section for the corresponding amounts of metal precursors and solutions and visual for the synthesis method). The mixture was then heated to 353 K with constant stirring until gel formation, followed by drying at 363 K for 24 h in air. The dried samples were pulverized to obtain a fine powder and calcined at 973 K in air for 5 h.

**Scheme 1:**
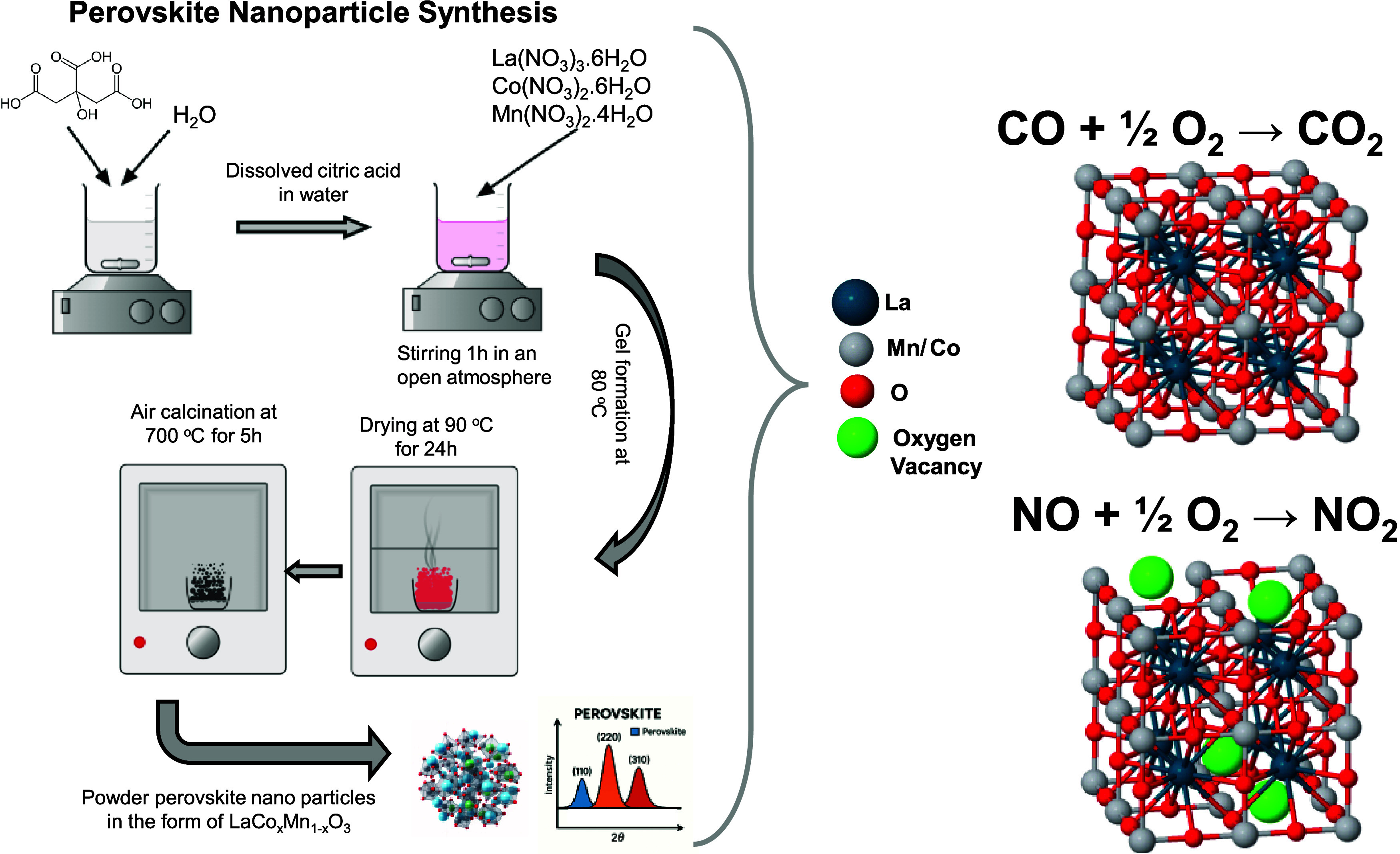
Citrate-Gel Synthesis Route for LaCo_*x*_Mn_1–*x*_O_3_ Perovskite Nanoparticles and Investigated Catalytic Reactions

A 1 wt % Pt/ γ-Al_2_O_3_ benchmark catalyst was synthesized using the incipient wetness impregnation method. γ-Alumina support was placed in a two-neck Erlenmeyer flask that is connected to a vacuum pump and a peristaltic pump. The sealed flask was placed in a sonicator for homogeneous distribution of the Pt precursor. The support material was outgassed at 0.2 atm for 30 min to evacuate the pores of the material prior to the Pt precursor injection. 480 μL of Pt­(NH_3_)_2_(NO_2_)_2_ in 1.5 mL of deionized water was added using the peristaltic pump with a 0.1 mL/min injection rate, followed by excess solvent removal under vacuum for 40 min. The resulting slurry was dried at 353 K for 3 h and calcined in air at 823 K (with a heating ramp of 2 K/min) for 3 h.

### 2.3 Catalytic Tests

NO and CO oxidation performance tests were conducted using a custom-designed plug-flow reactor system at atmospheric pressure (Figure S1). In the NO (CO) oxidation tests, 30 mg (40 mg) of a catalyst with a 45–60 mesh size was mixed with 420 mg (210 mg) of the α-Al_2_O_3_ diluent with a 45–60 mesh size and placed into a tubular quartz reactor (0.8 cm inner diameter, 47 cm long). A chemiluminescent NO_*x*_ analyzer (Teledyne T200) and a quadruple mass spectrometer (QMS; Stanford Research Systems, RGA200) were utilized for the quantitative real-time detection of NO/NO_2_ and CO/CO_2_ concentrations in the reactor outlet with a monitoring rate of 1 Hz for NO and CO oxidation reactions, respectively. In the NO oxidation tests, the reactor outlet gas was diluted with N_2_ (i.e., *outlet gas:N*_*2*_ = 1:47) and fed to the NO_*x*_ analyzer to maintain the NO_*x*_ concentration below 20 ppm, which is the upper detection limit of the NO_*x*_ analyzer. Experimental details of the catalytic test setup, reaction conditions, feed gas concentrations, and pretreatment parameters are provided in SI Section 2 and Figures S2 and S3.

### 2.4 Structural Characterization

X-ray diffraction (XRD) experiments were carried out with an X-ray diffractometer (PANalytical) equipped with a Cu Kα (1.5405 Å) X-ray source operating at 45 kV and 40 mA. Bright-field transmission electron microscopy (TEM), high-angle annular dark-field scanning transmission electron microscopy (HAADF-STEM), and energy-dispersive X-ray (EDX) analysis measurements were performed via a transmission electron microscope (FEI Tecnai G2F30). Brunauer–Emmett–Teller (BET) specific surface area (*S*_BET_, m^2^ g^–1^) measurements were executed via low–temperature isothermal adsorption–desorption of N_2_ using a Micromeritics TriStar 3000 apparatus. X-ray photoelectron spectroscopy (XPS) measurements were performed using a PHOIBOS MCD-9 hemispherical energy analyzer and a monochromatic Al Kα X-ray irradiation (*h*ν = 1486.74 eV, 350 W). During the XPS measurements, powder catalyst samples were placed on copper tape, and charge compensation was achieved using an electron flood gun (SPECS FG-15/40, 3.0 eV, 70 μA). Due to differential charging issues and complexity of the C 1s signal (owing to residual carbonaceous surface species originating from the citrate-based perovskite synthesis method), XPS binding energy (B.E.) positions were calibrated using the La 3d_5/2_ signal at 834.7 eV corresponding to the La^3+^ state. Bulk elemental compositions were measured using ICP-MS (Agilent 7700x). Prior to the ICP-MS measurements, each perovskite catalyst was dissolved in 2% (w/w) HNO_3_ (aq) and sonicated for 10 min. The nominal concentration of each sample solution was set to 5 ppm (metal basis). For the ICP-MS analysis of each perovskite, two different samples were taken, and each sample was measured five times. The standard deviations in ICP-MS measurements were found to be smaller than 1%.

### 2.5 Hydrogen Temperature-Programmed Desorption (H_2_-TPR) Experiments

In the H_2_-TPR experiments, 125 mg of a perovskite sample with a 45–60 mesh size was loaded into the flow reactor bed. Each catalyst was first pretreated in a 4% H_2_/Ar flow at 773 K for 1 h with a total flow rate of 500 mL (STP) min^–1^. The gas feed was then switched to pure Ar for 1 h at 773 K at the same flow rate. The final step of the pretreatment involved flowing 20% O_2_/Ar for 1 h at 773 K at a flow rate of 500 mL (STP) min^–1^. The catalyst was then cooled to 300 K in a 20% O_2_/Ar mixture without changing the flow rate. This pretreatment procedure was carried out to ensure surface cleaning and the removal of adsorbed carbonaceous synthesis residues (i.e., −C_*x*_H_*y*_O_*z*_(ads)) as well as molecular water from the catalyst surfaces.

After the completion of the pretreatment, the gas feed was switched to a 4% H_2_/Ar flow at a total flow rate of 500 mL (STP) min^–1^ for 4 h at 300 K to stabilize the H_2_O (*m*/*z* = 18) signal in a quadruple mass spectrometer (QMS). Once the QMS water signal reached a steady state, the catalyst was heated to 1173 K at a heating rate of 6 K.min^–1^ in the presence of 4% H_2_/Ar with a flow rate of 500 mL (STP) min^–1^. The reduction process was monitored by recording the QMS signal intensity of H_2_O (*m*/*z* = 18) as a function of temperature.

### 2.6 X-ray Absorption Near Edge Spectroscopy (XANES) Measurements

*Ex-situ* XANES measurements of as-prepared perovskites were conducted at the P65 Beamline of the DESY Petra III Synchrotron facility (Hamburg, Germany) and at the SAMBA beamline of the SOLEIL Synchrotron facility (Paris, France). *In situ* XANES measurements were carried out exclusively at the SAMBA beamline at various temperatures (393–973 K with a 10 K/min linear temperature ramp rate) in the presence of H_2_ (5% H_2_/He, 50 mL/min) or O_2_ (10% O_2_/He, 50 mL/min) using an *in situ* glass capillary tube reactor. XANES experiments were performed for the Co-K (7709 eV), Mn-K (6539 eV), and La-L-III (5483 eV) edges in transmission and/or fluorescence modes. For each measured sample, at least two subsequent spectra were acquired to enhance the signal-to-noise ratio (S/N). Data analysis was performed using the ATHENA software.^25^

### 2.7 *In Situ* Fourier Transform Infrared (FTIR) Spectroscopy Measurements

*In situ* FTIR spectroscopic measurements on NO_2_ disproportionation over perovskite samples were performed in transmission mode using a batch-type spectroscopic reactor equipped with an FTIR spectrometer (Bruker, Tensor 27), and a mercury–cadmium–telluride (MCT) detector. This *in situ* FTIR system was also coupled to a QMS (Stanford Research Systems, RGA200) for temperature-programmed desorption (TPD) experiments.^26^ Before the *in situ* FTIR spectroscopy experiments, samples were outgassed in the spectroscopic batch reactor under vacuum (<10^–3^ Torr) at 403 K for 12 h to clean/outgas the catalyst surface. The catalyst surfaces were then exposed to 0.5 Torr NO_2_ for 5 min at 323 K, followed by evacuation to <10^–3^ Torr and annealing in vacuum using a linear heating ramp (12 K/min) from 323 to 973 K for further oxidative cleaning of the surface. Details regarding NO_2_(g) preparation and purification can be found elsewhere.^26^

Next, NO_2_ adsorption was performed by dosing 5.0 Torr of NO_2_ over the sample for 10 min at 323 K, and *in situ* FTIR spectra were acquired on these fresh catalyst surfaces at 323 K after evacuation. In complementary FTIR spectroscopic experiments, clean perovskite surfaces were reduced in the presence of 5 Torr of H_2_ at 623 K for 10 min in the FTIR batch reactor. These prereduced perovskite surfaces were then exposed to 5.0 Torr of NO_2_ for 10 min at 323 K, and *in situ* FTIR data were acquired after evacuation at 323 K. An identical mass of (20 mg) catalyst was used in all *in situ* FTIR spectroscopic experiments for IR intensity comparison among investigated samples.

### 2.8 NO_*x*_-TPD Measurements

NO_*x*_-TPD experiments were carried out in vacuum, immediately after the i*n-situ* FTIR spectroscopic NO_2_ adsorption experiments. A heating ramp rate of 12 K/min was used in the TPD experiments. The following *m*/*z* desorption channels were simultaneously monitored during TPD: 28 (N_2_), 30 (NO/N_2_O/NO_2_), 44 (N_2_O), and 46 (NO_2_). Quantification of each desorbing gas phase species was calculated using corresponding experimental QMS fragmentation factors. Blank experiments (data not shown) revealed that CO­(g) and CO_2_(g) had negligible contributions to the *m*/*z* = 28 and *m*/*z* = 44 desorption channels, respectively.

## 3 Results

### 3.1 Structural Characterization of the Synthesized Perovskite Catalysts

XRD was employed to confirm the formation of LCM (i.e., LaCo_*x*_Mn_1–*x*_O_3_) rather than separate phases of LaMnO_3_ and LaCoO_3_. For reference, XRD patterns of the separately synthesized LaMnO_3_ and LaCoO_3_ structures were also obtained, showing the formation of cubic (PDF Card No. 04-006-9518) and rhombohedral (PDF Card No. 04-012-5614) phases, respectively (Figure 1a,b). XRD data illustrated that tuning the nominal Co/Mn cation ratio in the LCM induced phase transformations. With increasing Co loading, the cubic phase of the LaMnO_3_ structure transitioned to an orthorhombic form (PDF Card No. 04-012-5614) for LaCo_0.6_Mn_0.4_O_3_. A further increase in cobalt content (i.e., *x* ≥ 7) led to the formation of a rhombohedral structure (Scheme 2) similar to that of LaCoO_3_ (Figure 1a,b). The gradual formation of distinct crystal structures different from those of LaMnO_3_ and LaCoO_3_ simple perovskites indicates that the synthesized materials were in the form of LCM with varying Co/Mn ratios on the B-site rather than separate domains of LaMnO_3_ and LaCoO_3_. This argument is further supported by surface elemental (EDX) mapping analysis of the B-site cations on the LCM catalysts (Figure 1c–e) discussed in the forthcoming section.

**Figure 1:**
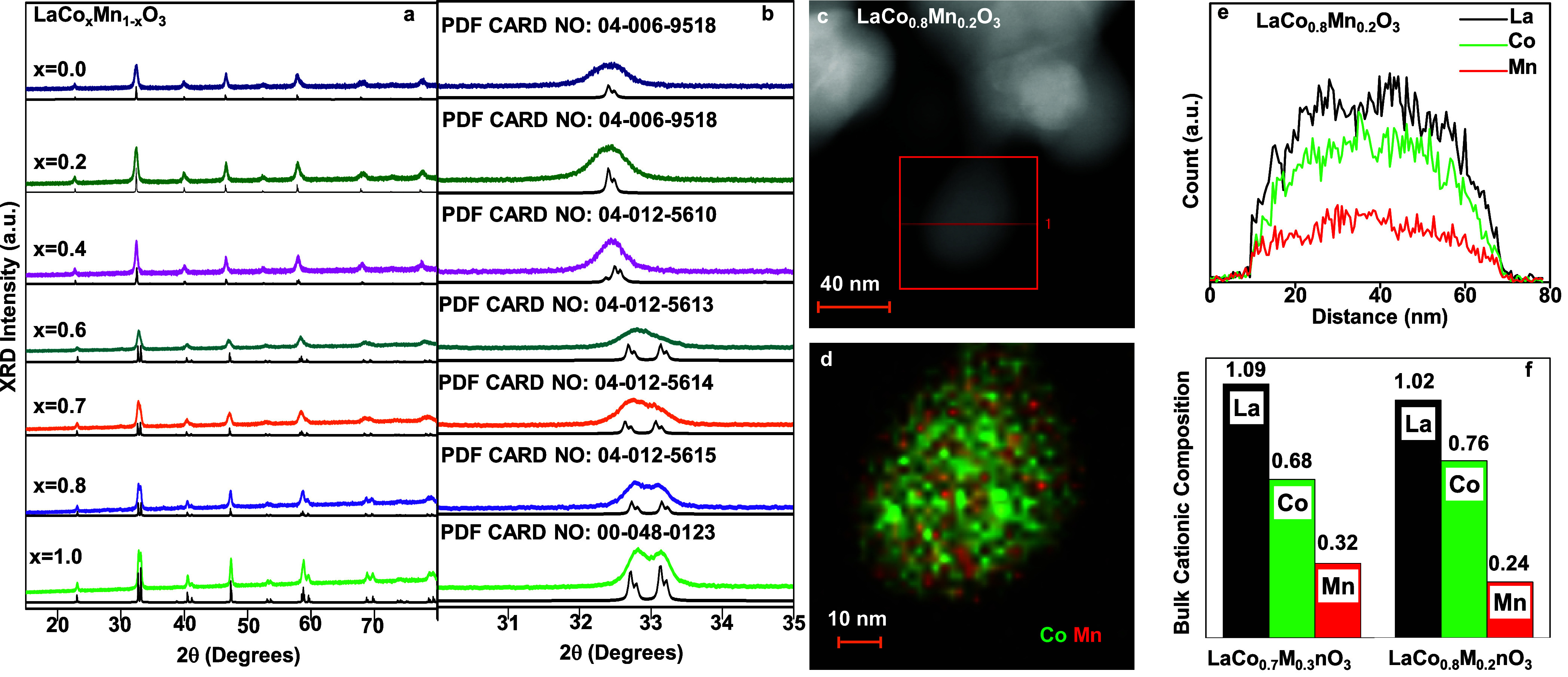
(a) XRD patterns within (a) 10° < 2θ < 80° and (b) 31° < 2θ < 35° of LaCo_*x*_Mn_1–*x*_O_3_. Black curves below each XRD pattern show the reference ICDD XRD data corresponding to the particular LaCo_*x*_Mn_1–*x*_O_3_ structures. (c) HAADF-STEM image of a LaCo_0.8_Mn_0.2_O_3_ particle. (d) Co and Mn EDX mapping, and (e) La, Co, and Mn EDX line scans of the LaCo_0.8_Mn_0.2_O_3_ particle given in (c). (f) Relative bulk cationic compositions of LaCo_0.7_Mn_0.3_O_3_ and LaCo_0.8_Mn_0.2_O_3_ based on ICP-MS. All samples correspond to fresh catalysts obtained after calcination at 973 K.

**Scheme 2:**
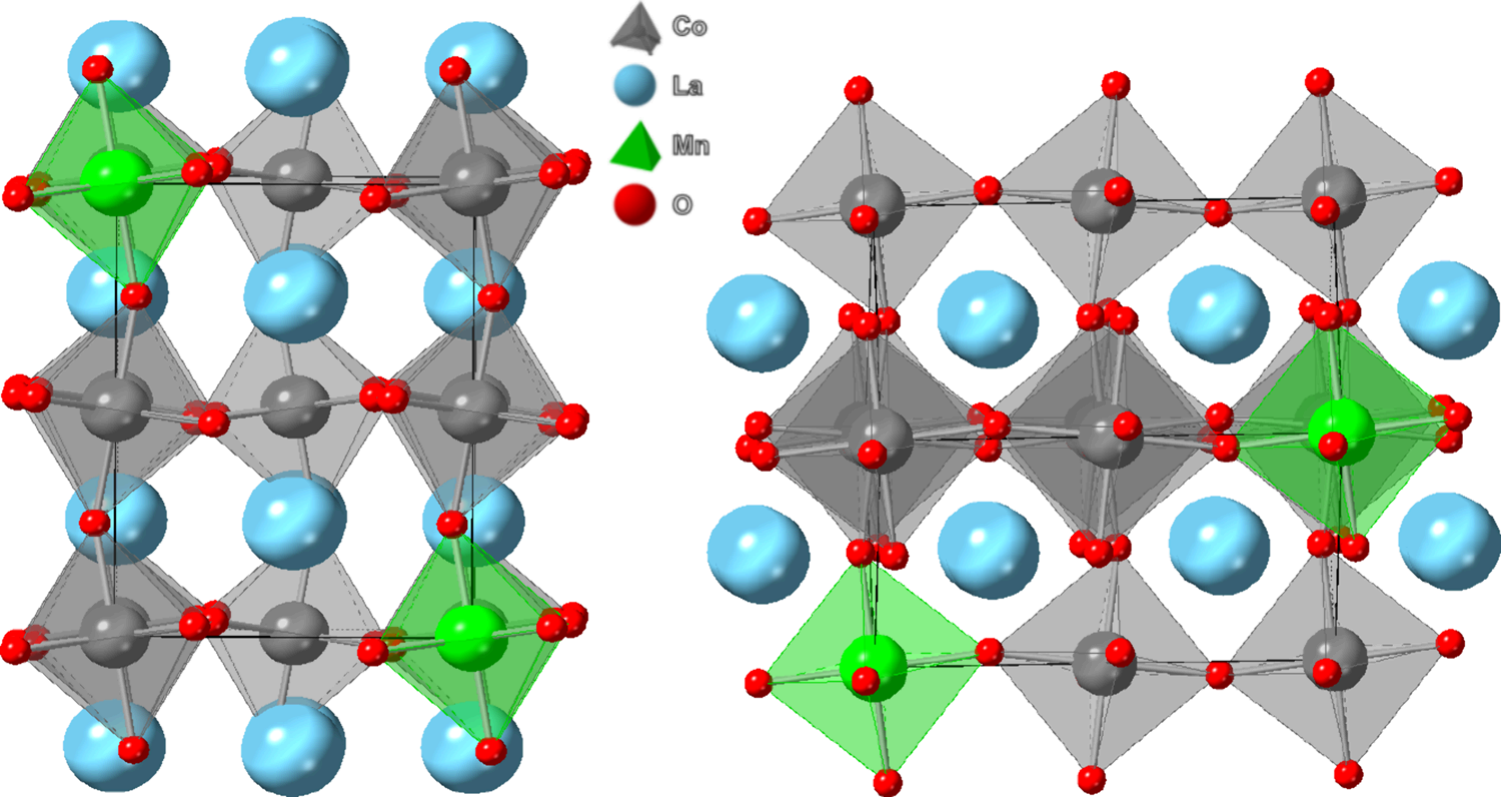
Generic Rhombohedral (*R*3*c*) Crystal Structure of LaCo_*x*_Mn_1–*x*_O_3_ Catalysts for *x* ≥ 0.7

Further support for the formation of LCM structures is provided by TEM-EDX and BET measurements. The HAADF image in Figure 1c reveals that the LaCo_0.8_Mn_0.2_O_3_-mixed perovskite particles have a diameter of approximately 30–40 nm (see Figure S4 for additional bright-field TEM images of the LaCo_0.7_Mn_0.3_O_3_ particles). EDX maps for a LaCo_0.8_Mn_0.2_O_3_ particle show that Co and Mn cations are rather uniformly distributed throughout particle (Figure 1d), in accordance with the lack of separate LaCoO_3_ and LaMnO_3_ domains. The EDX intensity profiles of La, Co, and Mn for the line scan across the particle shown in Figure 1c also indicate a homogeneous distribution of La, Co, and Mn (Figure 1e) throughout the LaCo_0.8_Mn_0.2_O_3_ nanoparticle. In addition, the specific surface area (SSA) measured for both LaCo_0.7_Mn_0.3_O_3_ and LaCo_0.8_Mn_0.2_O_3_ LCM samples was 16 m^2^/g, which is different than that of LC and LM (i.e., 8 and 21 m^2^/g, respectively, Figure S5).

ICP-MS data (Figure 1f and Table S2) show a reasonable correlation between the experimentally measured bulk cation compositions and the nominal metal precursor mole ratios used in the synthesis. Two of the best-performing LCM catalysts, namely, LaCo_0.8_Mn_0.2_O_3_ and LaCo_0.7_Mn_0.3_O_3_, were selected for ICP-MS analysis. The detectable differences between the measured Co and Mn compositions, despite very close metal loadings, suggest that the current synthetic protocol provides a high level of compositional tunability, which is crucial for optimizing catalytic performance. Note that while bulk stoichiometric compositions of the synthesized LC, LM, and LCM structures were comparable to those of nominal compositions expected from the relative initial amounts of metal precursors used in the synthesis, relative surface atomic composition measurements obtained from the current XPS data (Figure S6) suggested an enrichment of La and Mn species on the surface. Thus, presence of La-, Co-, and/or Mn-containing metal oxide, metal hydroxide, or metal oxyhydroxide minority domains on the synthesized LCM surfaces cannot be ruled out.

### 3.2 Catalytic CO and NO Oxidation Performances

The catalytic performances of the synthesized LC, LM, and LCM samples were evaluated in NO and CO oxidation reactions and compared to that of the 1 wt % Pt/Al_2_O_3_ benchmark catalyst (Figure 2). In the NO oxidation reaction, both simple perovskites and LCM catalysts could outperform the Pt/Al_2_O_3_ benchmark catalyst based on the NO conversion %, which increased with temperature (Figure 2a) and eventually converged to equilibrium values (i.e., dashed line in Figure 2a), in agreement with the former reports.^9^ Among the currently investigated perovskites, LaCo_0.8_Mn_0.2_O_3_ exhibited the highest NO conversion at the lowest temperature, reaching 62% at 626 K, whereas the Pt/Al_2_O_3_ catalyst did not achieve the equilibrium conversion even at 720 K. Similarly, LCM demonstrated higher activity in CO oxidation compared to the Pt/Al_2_O_3_ benchmark catalyst (Figure 2b). While 100% CO conversion was achieved for all catalysts except LaMnO_3_ within the studied temperature range, LaCo_0.8_Mn_0.2_O_3_ showed the lowest temperature for 50% CO conversion (i.e., *T*_50_ = 420 K). Note that control experiments carried out using solely α-Al_2_O_3_ (which was used as a diluent in the perovskite performance tests) resulted in negligible activity in both CO and NO oxidation reactions (Figures 2a,b). Consequently, LaCo_0.8_Mn_0.2_O_3_ demonstrated the highest catalytic performance in both NO and CO oxidation reactions among the tested catalysts.

**Figure 2:**
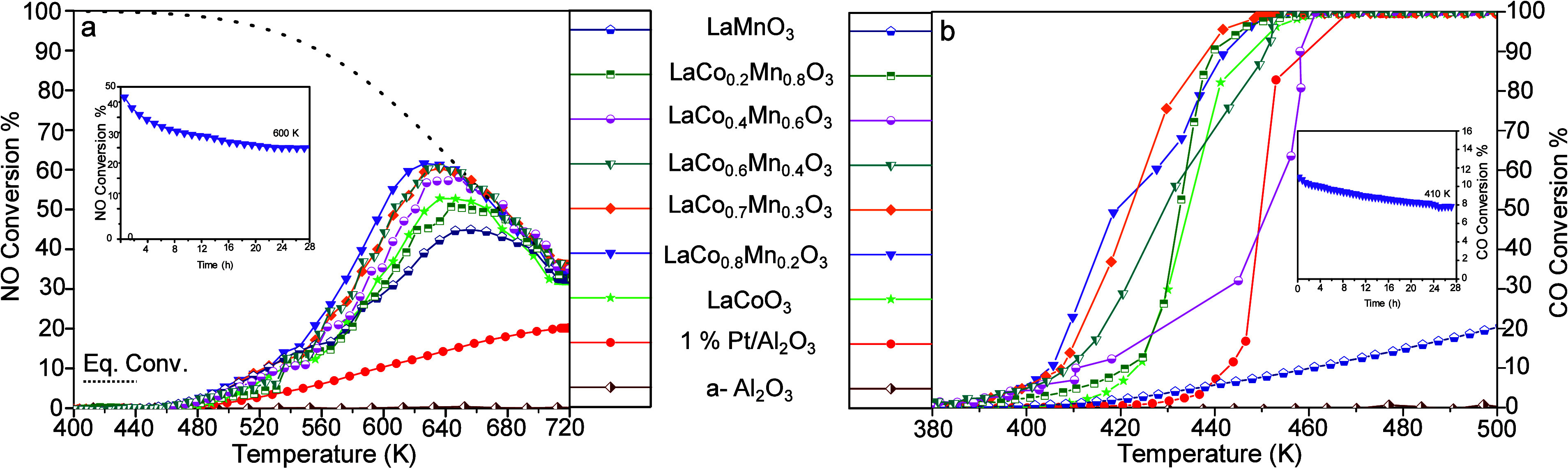
Catalytic (a) NO oxidation (700 ppm of NO­(g) and 8% O_2_(g) in Ar­(g), total flow rate = 500 mL (STP) min^–1^, heating rate 6 K min^–1^, GHSV = 50,000 h^–1^); top curve with black dashed line represents the equilibrium NO conversion at the given temperatures, which is calculated through the Gibbs free energy minimization approach at given concentrations. (b) CO oxidation (1.6% CO­(g) and 20% O_2_(g) in Ar­(g), total flow rate = 500 mL (STP) min^–1^, heating rate 4 K min^–1^, GHSV = 100,000 h^–1^) test results for LaCoO_3_, LaMnO_3_, LaCo_*x*_Mn_1–*x*_O_3_, and 1 wt % Pt/Al_2_O_3_ PGM benchmark catalyst. Insets in (a) and (b) show the extended (28 h) catalytic stability test results for LaCo_0.8_Mn_0.2_O_3_. Information regarding detailed experimental procedures and parameters can be found in SI Section 2.

The dissimilar line shapes in the conversion profiles of NO and CO oxidation reactions given in Figure 2a,b, along with the significantly different temperatures at which maximum conversion is achieved (626 K for NO and *T* ≥ 460 K for CO), suggest distinct reaction mechanisms and activation routes for these two oxidation processes on LCM surfaces. For the Pt/Al_2_O_3_ benchmark catalyst, both CO and NO oxidation reactions are expected to proceed exclusively via Langmuir–Hinshelwood (LH) type mechanisms. This is because Al_2_O_3_ is a nonreducible metal oxide, which is incapable of forming oxygen vacancies that are necessary for Mars-Van Krevelen (MvK)-type catalytic mechanisms.^27,28^ In contrast, as will be demonstrated via current *in situ* spectroscopic results, we propose that on the LCM catalysts, the LH-type mechanism is predominant for CO oxidation, whereas NO oxidation follows prevalently a MvK-type mechanism.

Former studies in the literature^9,20,29–31^ as well as the results that are presented in the forthcoming sections of this work (Figures 3, 4, and 5) clearly indicate that oxygen vacancy formation is rather unlikely to occur to a significant extent on LCM, LC, or LM catalysts below 520 K. Therefore, an MvK-type mechanism is not expected to be prevalently governing neither NO oxidation nor CO oxidation reactions below 520 K on the currently tested perovskites. CO conversion reaches to 100% below 460 K on all of the samples, suggesting that the LH-type mechanism is likely to be dominant for CO oxidation, which is consistent with former reports in the literature.^20,29^ In the case of NO oxidation, NO conversion % increases rather slowly as a function of increasing temperature up to ca. 550 K, followed by a readily recognizable break point in the NO conversion at 550 K, later followed by a much steeper rise at *T* ≥ 550 K, presumably due to a drastic change in the reaction mechanism at ca. 550 K.

**Figure 3:**
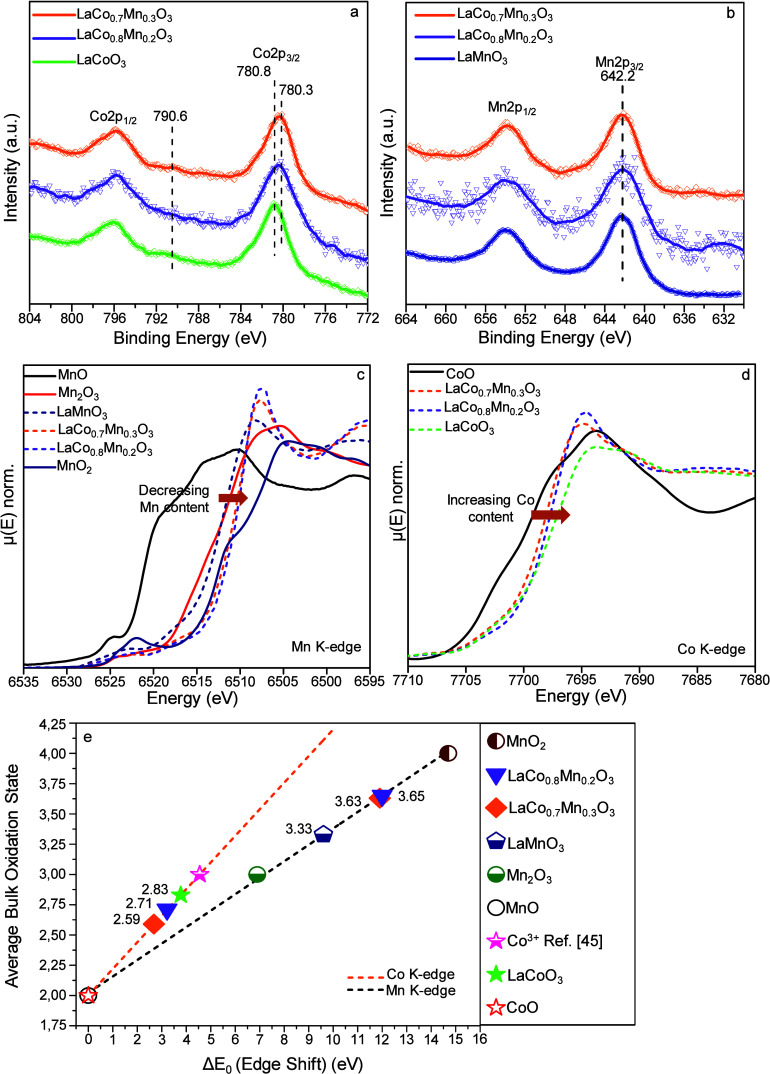
(a) Co 2p and (b) Mn 2p XPS, (c) Mn K-edge, and (d) Co K-edge XANES data for the synthesized LaCoO_3_, LaMnO_3_, LaCo_0.7_Mn_0.3_O_3_, and LaCo_0.8_Mn_0.2_O_3_ catalysts, as well as the corresponding metal oxide benchmark materials. (e) Calibration curves for oxidation states of Co and Mn cations generated using the K-edge energy shifts of the corresponding reference materials in XANES (see SI Sections 3–5 for details).

**Figure 4:**
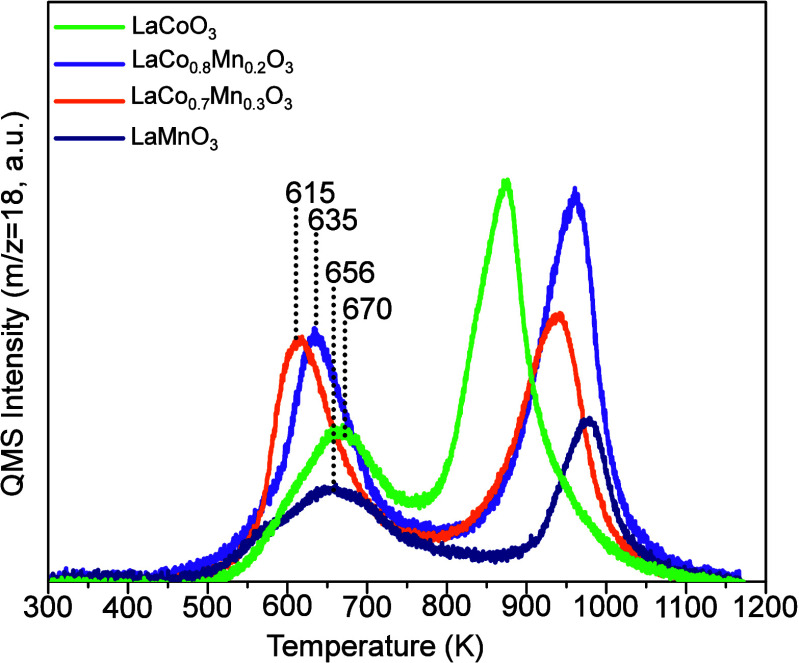
H_2_-TPR profiles for LaMnO_3_, LaCoO_3_, LaCo_0.7_Mn_0.3_O_3_, and LaCo_0.8_Mn_0.2_O_3_ catalysts.

**Figure 5:**
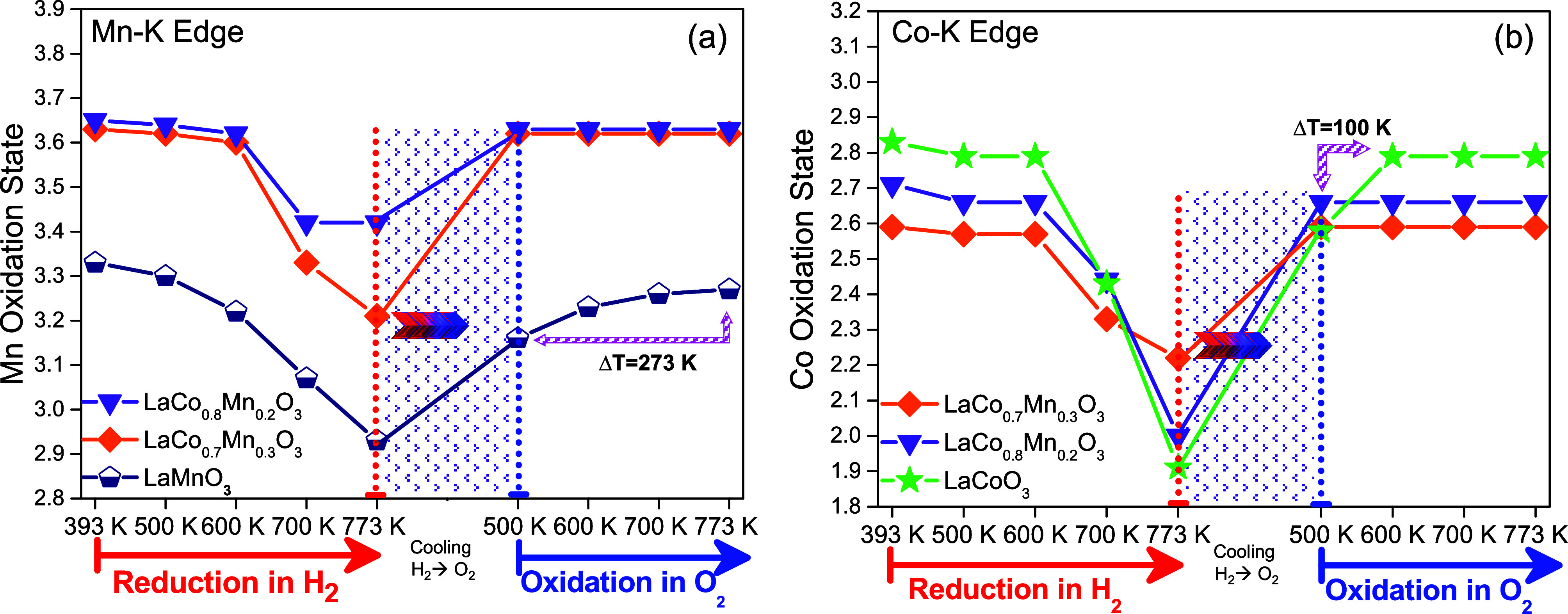
In situ XANES data for (a) Mn and (b) Co bulk oxidation states of LaMnO_3_, LaCoO_3_, LaCo_0.7_Mn_0.3_O_3_, and LaCo_0.8_Mn_0.2_O_3_ acquired during consecutive reduction with H_2_ (g) flow and oxidation with an O_2_ (g) flow at various temperatures.

Extended (28 h) isothermal catalytic durability tests were also conducted for the best-performing LaCo_0.8_Mn_0.2_O_3_ catalyst in both NO and CO oxidation reactions (Figure 2a,b, insets). NO oxidation was performed at 600 K, where the initial NO conversion was 50%. For CO oxidation, a lower temperature of 410 K (30% initial CO conversion) was chosen to mitigate local heat buildup due to the exothermic nature of the reaction. Although the initial CO and NO conversion % values somewhat decreased over extended reaction durations, the LaCo_0.8_Mn_0.2_O_3_ perovskite managed to reveal reasonable stability and durability during the CO and NO catalytic oxidation processes within 28 h.

### 3.3 Electronic Structures of the A and B Sites of the Perovskites

Surface electronic structures of the currently studied perovskites were examined via XPS, while bulk average oxidation states of Co and Mn sites were investigated via XANES measurements (note that the typical surface sensitivity of the XPS technique is ca. <10 nm,^32^ while that of XANES is ca. <200 nm).^33,34^

XPS analysis revealed a B.E. shift of −0.5 eV for the main Co 2p_3/2_ signal in LaCo_0.8_Mn_0.2_O_3_ (780.3 eV) and LaCo_0.7_Mn_0.3_O_3_ (780.3 eV), as compared to that of the LC (780.8 eV) catalyst (Figure 3a). In good agreement with former reports,^31,35,36^ the main Co 2p_3/2_ signal and the small satellite at 790.6 eV of the LC catalyst can be predominantly attributed to the Co^3+^ sites of the perovskite phase, while the −0.5 eV shift in Co 2p_3/2_ signal of LCM can be associated with the existence of the Co^2+^ state, similar to those found in Co_3_O_4_ (the convoluted Co 2p_3/2_ B.E. signal for Co_3_O_4_ typically appears at ca. 779.6 eV).^37–39^ Note that the presence of CoO or Co­(OH)_2_ species on LC or LCM sample surfaces seems to be unlikely due to the lack of the characteristic strong Co 2p satellite feature that should be observed at ca. 786 eV.^39^

The presence of reduced Co sites, indicated by a negative shift of 0.5 eV in Co 2p_3/2_ B.E. on the LCM sample surfaces, can be attributed to a relative enrichment in the electron density of the Co sites, which is likely facilitated by an electron density transfer from Mn sites toward Co sites. Observation of a lower Co oxidation state in the LCM as compared to simple perovskites could be significant in oxidation reactions, as it was reported in a former study that O_2_ adsorption is stronger on Co^2+^ sites than that of Co^3+^ sites.^40^ In other words, O_2_ adsorption strength and the surface residence time of O_2_ (ads) are expected to be greater on the LCM surfaces, revealing Co sites with lower oxidation states facilitating reactant stabilization on the catalyst surface.

The Mn 2p_3/2_ binding energy of 642.2 eV in LM is associated with Mn^4+^ states (Figure 3b).^41,42^ No detectable B.E. shift was observed for the Mn 2p_3/2_ signal in the LCM samples as compared to that of LM, most likely due to the convoluted nature of the Mn 2p signal and the smaller B.E. shifts in the Mn 2p features that are too small to be resolved with the currently available XPS energy resolution.

Variation in the average Mn and Co bulk oxidation states of the LCM catalysts as a function of the nominal perovskite composition was also investigated through *ex-situ* XANES analysis. The edge energies of the B-site cations in the synthesized samples were measured relative to reference materials such as CoO, MnO, Mn_2_O_3_, and MnO_2_ with well-known Co or Mn oxidation states (Figure 3c,d and Table S3). To determine the bulk average oxidation states of Mn and Co in the mixed perovskites, the relative edge energies of the corresponding elements in the reference samples were plotted against their oxidation states, and linear calibration curves were obtained for both B-site metals (Figure 3e). A monotonic shift in the Mn and Co K-edge energies was observed as a function of varying B-site Co or Mn content. In contrast, no shift in the La L-III-edge energy was detected for the perovskite samples, indicating that La remains in the +3 oxidation state in all investigated samples (Figure S7).

Using these calibration curves, the average bulk oxidation states of Co in LC and Mn in LM were found to be +2.8 and +3.3, respectively, indicating the presence of mixed oxidation states (Co^2+^/Co^3+^ and Mn^3+^/Mn^4+^) in simple perovskites, in good agreement with the surface oxidation states obtained from the current XPS results (Figure 3a,b) as well as the former reports in the literature.^43,44^ Along these lines, the bulk average Co and Mn oxidation states for the best-performing LaCo_0.8_Mn_0.2_O_3_ perovskite were determined to be +2.7 and +3.7, respectively. It is apparent that doping Mn into LC decreases the bulk average oxidation state of Co, while Mn exhibits a more oxidic character compared to LM. These results reveal that the B-site electronic structure of the LCM catalysts in their bulk is notably different from that of the LC^45^ and LM simple perovskites, contributing to the enhanced catalytic performance observed for the LCM catalysts.

### 3.4 Reducibility of the Synthesized Perovskites

It is often postulated in the literature that the formation of oxygen vacancies in perovskites via reduction enhances their catalytic performances.^20,29^ Thus, both surface and bulk oxygen vacancy concentrations as well as the tendency of the B-site cations to facilitate the release of neighboring oxygen species in a catalytic cycle are critical factors influencing the overall catalytic performance.

To demonstrate the reducibility of the B-site cations as a function of temperature, H_2_-TPR experiments were performed on LC, LM, and the best-performing LCM catalysts (Figure 4). It is important to emphasize that as can be seen in Figure 4, no significant oxygen release is detectable for LC, LM, and LCM catalysts below 520 K. This observation strongly supports the lack of MvK-type reaction mechanisms for CO oxidation and NO oxidation reactions as the generation of oxygen vacancies is not likely to occur below 520 K even in the presence of an aggressive reducing agent such as H_2_ (note that such an aggressive reducing agent is not present in CO oxidation and NO oxidation reactions, which further precludes the formation of oxygen vacancies below 520 K in CO/NO oxidation reactions). Oxygen removal in H_2_-TPR was observed in two distinct temperature windows (i.e., 550–750 and 750–1050 K) for LC, LM, and LCM catalysts. As will be discussed later in this text, we argue that the moderate temperature (550–750 K) H_2_-TPR signal, which is relevant to the temperature range of the currently investigated NO and CO oxidation reactions, is likely to stem from the reduction of both surface and bulk sites, while the higher temperature (>750 K) reduction process is presumably associated with the removal of oxygen from the bulk of the perovskite structure and/or possibly to a lesser extent, due to dehydroxylation of the perovskite surfaces.

Figure 4 shows that within the temperature range pertinent to NO and CO oxidation reactions investigated in the current report (i.e., *T* < 650 K), LaCo_0.7_Mn_0.3_O_3_ and LaCo_0.8_Mn_0.2_O_3_ catalysts can be reduced at temperatures lower than that of simple perovskites. Hence, the H_2_-TPR peak positions of the LCM catalysts were found to be located at 35–55 K lower temperatures than those of LC and LM. Additionally, investigation of the relative H_2_-TPR peak areas of the LCM as compared to that of the LC and LM catalysts at *T* < 650 K reveals that the LCM release significantly more oxygen, indicating that the LCM possess a greater tendency to be reduced in the presence of a reducing agent, which is in good accordance with their superior catalytic performance of LCM in the CO and NO oxidation reactions as compared to that of simple perovskites (Figure 2).

In an attempt to investigate oxygen dynamics of the synthesized perovskite systems under reaction conditions in real time, bulk oxidation states of the B-site cations were monitored via *in situ* XANES during the oxygen removal under H_2_ (g) flow within the catalytically relevant temperature window (393–773 K) (Figure 5a,b; the corresponding XANES spectra are given in Figures S8 and S9). *In situ* XANES results presented in Figure 5a,b illustrate an insignificant variation in the Co and Mn oxidation states during H_2_ reduction at temperatures *T* ≤ 500 K in very good agreement with the H_2_-TPR results presented in Figure 4. On the other hand, a clearly visible decrease in the oxidation states of both Co and Mn species were observed within 500–773 K in all of the perovskite catalysts, coinciding with the low-temperature H_2_-TPR signals observed in Figure 4. Since the XANES technique predominantly probes bulk sites rather than the surface sites, it follows that the low-temperature H_2_-TPR signals observed in Figure 4 have some contribution from the reduction of the bulk sites in the perovskites. However, it should be noted that the existence of surface reduction processes within 500–773 K cannot be excluded, which will be further verified via the current *in situ* FTIR spectroscopic experiments presented in Figure 6.

**Figure 6:**
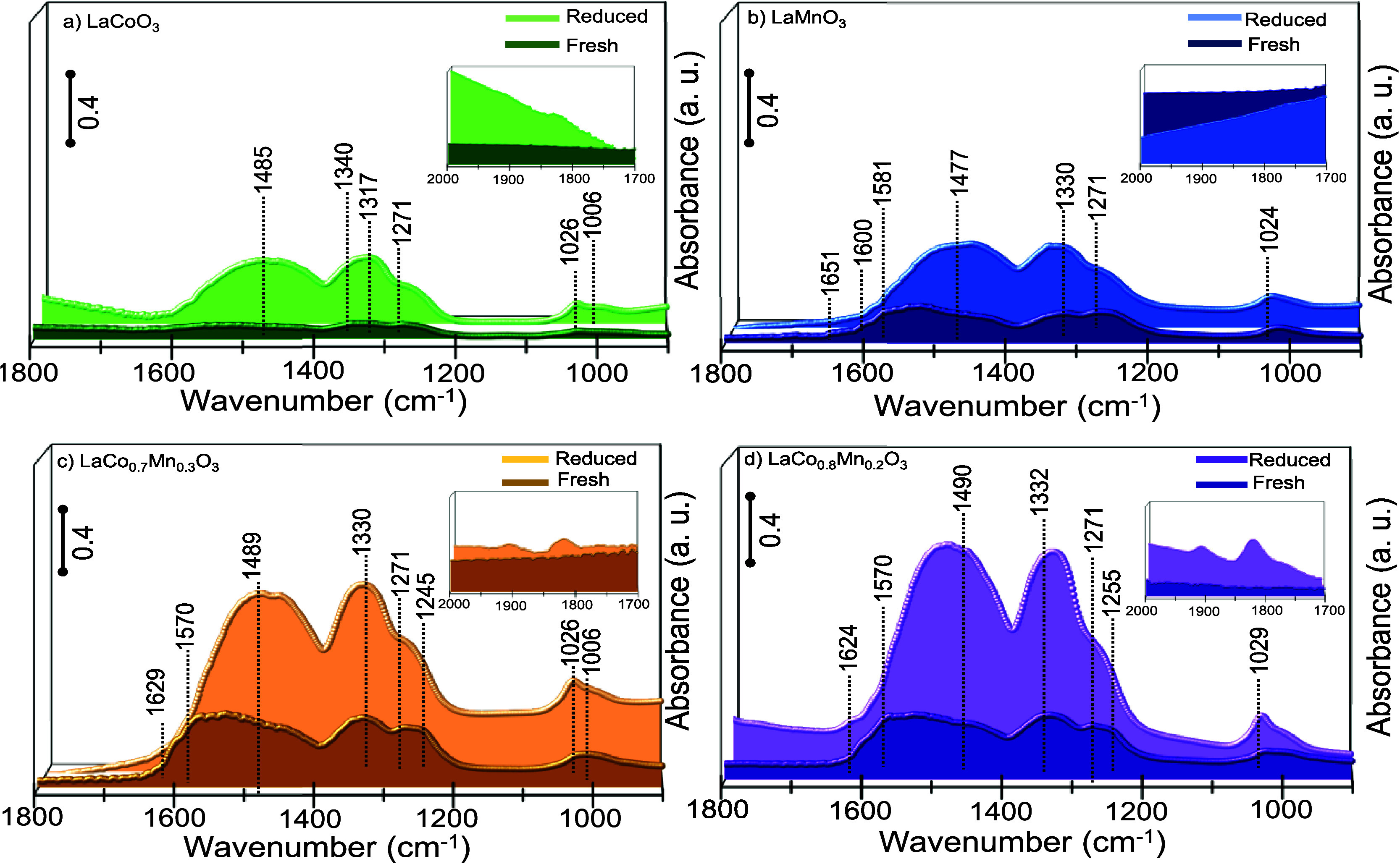
*In situ* FTIR spectra for NO_2_(g) disproportionation at 323 K on fresh and prereduced forms of (a) LaMnO_3_, (b) LaCoO_3_, (c) LaCo_0.7_Mn_0.3_O_3_, and (d) LaCo_0.8_Mn_0.2_O_3_ with H_2_ at 623 K.

Oxygen regeneration capability of the B-site cations and reversibility of the oxygen release and uptake processes under reducing or oxidizing reaction conditions are also critical factors influencing the oxidation reaction performance and catalytic longevity of the perovskites. In order to address this point, immediately after reducing the perovskite samples in H_2_(g) flow at 773 K, we switched the feed gas to O_2_(g) at 773 K and cooled the sample to 500 K in O_2_(g), followed by annealing in O_2_(g) up to 773 K (Figure 5), in an attempt to refill the oxygen vacancies with oxygen. *In situ* XANES data in Figure 5 showed that the LaCo_0.7_Mn_0.3_O_3_ and LaCo_0.8_Mn_0.2_O_3_ catalysts fully recovered their bulk Mn and Co oxidation states at a significantly lower temperature of 500 K and also after a relatively shorter duration of the O_2_(g) exposure as compared to that of simple perovskites. In contrast, LC and LM catalysts required higher temperatures of 600 and 773 K, respectively, to achieve full regeneration as well as a longer total duration of the exposure to O_2_(g) (note that since the *in situ* XANES data for different temperatures were acquired consecutively, data corresponding to higher temperatures also corresponded to longer total durations of the exposure to the O_2_(g) exposure). Accordingly, we argue that since an MvK-type reaction mechanism necessitates facile shuttling between reduced and oxidized states of the B sites of the catalyst through effective oxygen/vacancy dynamics, the enhanced redox regeneration capability of the LCM catalysts can render these materials superior in NO oxidation reaction at *T* > 550 K via an MvK-type mechanism.

After having investigated the bulk reducibility behavior of the synthesized perovskites, we examined the relative tendencies of the LCM versus LC and LM catalysts toward being reduced on the surface. Hence, relative surface reduction capabilities of the synthesized perovskites were studied via *in situ* transmission FTIR spectroscopy (Figure 6). Here, NO_2_ was exploited as a probe molecule to monitor the relative quantities of surface oxygen vacancy formation under the reducing conditions. Disproportionation reaction (1) of adsorbed NO_2_ species yielding nitrates (NO_3_^–^) and nitrosonium/nitrosyl (NO^+^/NO) species is well-known to be highly sensitive to the presence of surface oxygen vacancies. Therefore, NO_2_ (ads) can be used as an efficient probe to titrate surface oxygen vacancies through the detection of surface nitrate formation.^26,46^ Upon NO_2_ adsorption on reducible metal oxide surfaces, NO_2_ interacts with surface oxygen vacancies (* (surf)) to form NO^+^ (ads) (2)*.* Ionic surface oxygen species (O^–^ (ads)) generated due to reaction (2) can then react with adsorbed NO_2_ molecule to form NO_3_^–^ (ads) (3).^40^ Therefore, the relative coverage of surface nitrates (NO_3_^–^) on different perovskites upon NO_2_ adsorption can be used as an indicator to compare the relative capabilities of different perovskite surfaces to generate surface oxygen vacancies. Note that currently investigated perovskite samples strongly absorb mid-IR radiation within 2000–2400 cm^–1^ precluding the detection of NO^+^(ads) species, whose IR spectroscopic features (2100–2300 cm^–1^) fall within this frequency window. However, NO (ads) species could be observed in the inset plots of Figure 6. Due to the same material-based spectroscopic limitation, CO adsorption could not be investigated via in situ FTIR spectroscopy on the investigated perovskite samples.

$$2{\mathrm{NO}}_{2}\left(\mathrm{ads}\right)\to {\mathrm{NO}}_{3}^{-}\left(\mathrm{ads}\right)+{\mathrm{NO}}^{+}\left(\mathrm{ads}\right)$$ 1

$${\mathrm{NO}}_{2}(\mathrm{ads})+{\;}^{*}(\mathrm{surf})\to {\mathrm{NO}}^{+}(\mathrm{ads})+O^{-}(\mathrm{ads})$$ 2

$${\mathrm{NO}}_{2}(\mathrm{ads})+O^{-}(\mathrm{ads})\to {\mathrm{NO}}_{3}^{-}(\mathrm{ads})$$ 3

*In situ* FTIR spectra presented in Figure 6 shows the characteristic vibrational signals of NO_3_^–^ species in various adsorption geometries on all of the investigated perovskite surfaces upon NO_2_ adsorption at 323 K. These signals include symmetric stretching (ν_s_ = 970–1040 cm^–1^) and asymmetric stretching modes, where the latter set of modes split into two bands at high (ν_as_ = 1480–1650 cm^–1^) and low frequencies (ν_as_ = 1170–1300 cm^–1^), respectively. Based on the vast number of former *in situ* spectroscopic studies on NO_2_ and NO+O_2_ adsorption on numerous metal oxide/perovskite surfaces,^26,27,47^ we assign the vibrational features in Figure 6 to predominantly nitrate species.^48^ However, as can be seen in the vibrational spectroscopic assignment information provided in Table 1, the presence of minority nitrite species on the surface cannot be excluded. Comparison of the relative nitrate IR signal intensities for different types of perovskites in Figure 6 indicates a much higher surface coverage of nitrate species on the LCM surfaces both in their fresh and prereduced forms as compared to LC and LM, suggesting a higher surface density of oxygen vacancies on the LCM catalysts. It should be emphasized that the observation of relatively greater nitrate IR signals for LCM cannot be merely attributed to the corresponding specific surface area values of the investigated catalysts. As can be seen in Figure S5, specific surface area (SSA) values of different catalysts do not yield a direct correlation between the observed IR nitrate signals intensities given in Figure 6 and the corresponding SSA values. Note that all samples analyzed in Figure 6 via *in situ* FTIR spectroscopy had an identical mass of 20 mg.

**Table 1:** Infrared Vibrational Frequencies and Vibrational Mode Assignments (ν_s_, Symmetric Stretch and Asymmetric Stretch, ν_as_) for Nitrate and Nitrite Species with Various Adsorption Geometries on the Synthesized Perovskites^a^

	ν_s_	ν_as_
monodentate nitrate (−ONO_2_)	970 −1035	1250–1290/1480–1530
bidentate nitrate (−O_2_NO)	1010–1040	1260–1300/1500–1565
bridging nitrate ((−O)_2_NO)	1000–1030	1170–1225/1600–1650
monodentate nitrite (−ONO)	1050–1065	1450–1470
bridging nitrite ((−O)_2_N)		1205–1220
monodentate nitro −NO_2_)	1315–1350	1335–1440
chelating nitro ((−O) (−NO))	1180–1260	1390–1520

a Adapted from ref 48.

The same set of perovskite surfaces was also analyzed via *in situ* FTIR spectroscopy upon reduction by H_2_ at 623 K followed by exposure to NO_2_ at 323 K. A noticeable enhancement of the vibrational signal intensities of NO_3_^–^ species was observed on the prereduced surfaces, providing additional evidence for the correlation between the surface oxygen vacancy population and the NO_2_ disproportionation reaction. Overall, current *in situ* XANES and *in situ* FTIR results imply that while oxygen vacancies are predominantly generated on the surface at temperatures below 550 K, their formation in the bulk becomes more facile at higher temperatures.

Furthermore, results of the NO_*x*_-TPD experiments given in Figure S10, which were carried out immediately after the in situ FTIR experiments presented in Figure 6, quantitatively verify the greater amounts of NO_*x*_ adsorption and stabilization of NO_*x*_ species on pristine as well as reduced forms of LCM catalysts containing both Co and Mn sites as compared to that of LC and LM catalysts, which contain only one type of B-site cation (Co or Mn). The greater amount of NO­(g) desorption observed in the NO_*x*_-TPD experiments illustrated in Figure S10 can be attributed to the thermal decomposition of adsorbed nitrates and nitrites as well as direct desorption of NO (ads)/NO^+^ (ads) from the catalyst surface. Thus, it can be argued that in the NO oxidation reaction, the presence of Mn species on the LCM catalyst surfaces can presumably increase the adsorption strength of NO­(g), facilitating the total oxidation of NO into NO_2_.

## 4 Discussion

Catalytic reaction mechanisms operating during the NO oxidation and CO oxidation reactions on LC, LM, and LCM catalysts can now be discussed in light of the currently provided detailed characterization results (Scheme 3). It is clear that in the CO oxidation reaction, CO conversion can readily reach 100% at *T* ≤ 450 K on LCM (Figure 2b), where almost no oxygen release or perovskite reduction were detected (Figures 4 and 5). These observations strongly suggest that generation of oxygen vacancies is not necessarily a prerequisite in CO oxidation reaction to reach full conversion. Thus, on the currently investigated LCM catalysts, conventional Langmuir–Hinshelwood (LH)-type mechanisms can be predominantly operating during the CO oxidation reaction in an exclusive manner at *T* < 460 K.

**Scheme 3:**
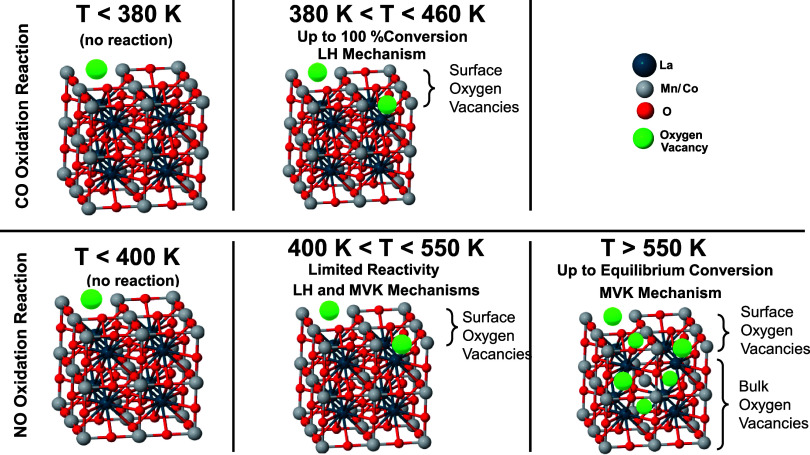
Catalytic Reaction Mechanisms Operating on the Currently Investigated LCM Catalysts at Different Temperatures^a^

a MvK and LH stand for Mars-Van Krevelen and Langmuir–Hinshelwood type reaction mechanisms, respectively.

In the NO oxidation reaction, conversion increases rather slowly with an increasing temperature within 380–550 K (Figure 2a). This is accompanied by a rather limited extent of surface and bulk reduction phenomena, as depicted in Figures 4 and 5. Therefore, within this thermal window, in addition to LH-type mechanisms, contribution of Mars-Van Krevelen (MvK)-type mechanisms (presumably in a minor fashion) to NO oxidation reaction on LCM catalysts cannot be excluded. On the other hand, at temperatures greater than 550 K, a clearly visible break point is observed in the NO conversion, where the NO oxidation rate significantly accelerates. This behavior coincides with the rapid generation of oxygen vacancies via surface and bulk reduction phenomena (Figures 4–6), implying that within 550–650 K, MvK-type mechanisms could be prevalently operating in NO oxidation on LCM catalysts.

An important point to address in the investigation of CO oxidation and NO oxidation reactions on the LCM catalysts is the individual catalytic functions of different types of B-site cations (i.e., Co and Mn). As discussed above, while the operating catalytic reaction mechanisms may differ for these two different reactions at various temperatures, both reactions share similar types of initial mechanistic steps, namely, adsorption and stabilization of reactants (i.e., CO/NO and O_2_) followed by activation of adsorbed O_2_ (note that particular CO and NO oxidation steps can be quite different in those two dissimilar reactions due to the involvement of oxygen vacancies and the formation of CO_2_, CO_3_^2–^, NO_2_, NO_2_^–^, NO_3_^–^, etc., species). Nevertheless, since the optimized LCM catalyst in NO oxidation and CO oxidation reactions was found to be LaCo_0.8_Mn_0.2_O_3_, revealing a Co-rich composition both on the surface as well as in the bulk (Figure S6 and Figure 1f), it can be argued that the primary catalytically active sites in both of these reactions are likely to be Co sites, while minority Mn sites function mainly as promoters. This argument is also consistent with the catalytic performance data given in Figure 2, where it can be clearly seen that among the simple perovskites, LaCoO_3_ is noticeably superior to LaMnO_3_, in both CO and NO oxidation reactions. Accordingly, in the case of the CO oxidation reaction, we propose that CO/O_2_ adsorption as well as O_2_ activation predominantly occur on Co sites. Furthermore, as discussed earlier, a relatively lower Co oxidation state observed for the surface and bulk of the LaCo_0.8_Mn_0.2_O_3_ catalyst as compared to that of LaCoO_3_ may also be responsible for the stabilization of O_2_ (ads) on the LaCo_0.8_Mn_0.2_O_3_ surface,^40^ expediting both CO oxidation and NO oxidation reactions.

On the other hand, the catalytic promotion effect of Mn sites can operate at least in two different ways. First, Mn sites can enhance the CO or NO adsorption on the catalyst surface by generating strongly bound carbonyls/carbonates^49–53^ or nitrosyls/nitrites/nitrates. Increase in the adsorption energy of CO has been frequently reported for MnO_*x*_-promoted Co/CoO_*x*_ catalysts in Fischer–Tropsch synthesis (FTS).^49–52^ In addition, a similar effect has also been observed in the formic acid dehydrogenation catalysts.^53^ It has been demonstrated that catalytic deactivation of PGM-based formic acid dehydrogenation catalysts due to CO poisoning can also be circumvented via MnO_*x*_ promotion, where MnO_*x*_ sites act as efficient CO-anchoring sites leading to the formation of strongly bound carbonyls and carbonates, thereby keeping PGM sites available for formic acid adsorption and dehydrogenation.^53^ Second, as demonstrated in our electron spectroscopic investigations presented in the current work, Mn addition to the LaCoO_3_ structure alters the oxidation state of the Co sites and results in an enrichment in the electron density of the Co sites in the optimized LaCo_0.8_Mn_0.2_O_3_ catalyst. It is possible that this electronic promotional effect can also positively influence O_2_ activation on the LaCo_0.8_Mn_0.2_O_3_ catalyst surface, which is consistent with the facilitated refilling of the oxygen vacancies of the LaCo_0.8_Mn_0.2_O_3_ catalyst by O_2_ at lower temperatures than those of LC and LM catalysts (Figure 5).

Since NO oxidation is predominantly governed by MvK-type mechanisms, it is also presumable that the presence of Mn sites with various oxidation states and a variety of ionic radii in the LaCo_0.8_Mn_0.2_O_3_ structure can further increase the number of lattice/surface imperfections/oxygen vacancies and facilitate the refilling of oxygen vacancies, which in turn may result in enhanced O_2_ activation as well as stronger NO_*x*_ adsorption (Figure 6).

## 5 Conclusions

Precious metal-free mixed B-site perovskite nanoparticles in the form of LaCo_*x*_Mn_1–*x*_O_3_ (LCM) with various Co:Mn atomic ratios and their simple perovskite counterparts, (i.e., LaCoO_3_ and LaMnO_3_, LC and LM) were also synthesized in the form of nanoparticles and investigated as catalysts in the CO oxidation and NO oxidation reactions. Our major findings can be summarized as follows:

i) The highest catalytic activities in both CO oxidation and NO oxidation reactions were observed for a Co-rich LCM catalyst with the nominal composition of LaCo_0.8_Mn_0.2_O_3_, surpassing all other currently investigated perovskites as well as a 1 wt.% Pt/Al_2_O_3_ PGM benchmark catalyst.
ii) LaCo_0.8_Mn_0.2_O_3_ yielded 100% CO conversion at *T* ≥ 460 K and revealed the highest NO conversion at the lowest temperature, reaching 62% at 626 K.
iii) CO oxidation proceeds exclusively via Langmuir–Hinshelwood (LH)-type mechanisms on the investigated perovskites at *T* < 460 K. On the other hand, at *T* < 550 K, NO oxidation reaction is likely to follow mostly LH-type catalytic reaction mechanisms with a minor contribution from Mars-van Kravelen (MvK)-type reaction mechanisms, while at temperatures above 550 K, MvK-type mechanisms become prevalent in the NO oxidation reaction.
iv) Adjustment of the B-site cation (Co:Mn) ratio in the LCM perovskite nanoparticle system allowed fine-tuning of the electronic structure of the B-site cations. In the optimized LaCo_0.8_Mn_0.2_O_3_ LCM nanoparticle catalyst, Co sites were found to be enriched in electron density, while Mn sites were found to be more electron deficient as opposed to LC and LM, respectively. Co^3+^ and Mn^4+^ species were found to be the dominant B-site cations both on the surface as well as in the bulk of the LaCo_0.8_Mn_0.2_O_3_ catalyst, while Co^2+^ and Mn^3+^ species existed as minority species.
v) LaCo_0.8_Mn_0.2_O_3_ not only released significantly greater amounts of oxygen and generated larger amounts of oxygen vacancies than that of LC and LM under reducing conditions but also managed to achieve this at lower temperatures, demonstrating a greater tendency toward being reduced, which is an essential characteristic required by MvK-based reaction mechanisms.
vi) Redox reversibility of the B-site cations (i.e., Co and Mn) and the regeneration of oxygen vacancies were shown to be much more facile for LaCo_0.8_Mn_0.2_O_3_, as opposed to simple perovskite nanoparticles. This is in line with the favorable extended catalytic activity (28 h) and stability of the LCM catalysts, requiring reversible formation and refilling of oxygen vacancies and associated reversible changes in the B-site oxidation state.
vii) It was proposed that the prominent active site in the CO oxidation and NO oxidation reactions on the LaCo_0.8_Mn_0.2_O_3_ perovskite catalyst are the Co cationic sites, while oxygen vacancies can also take part in the NO oxidation reaction. It is likely that Co sites on LaCo_0.8_Mn_0.2_O_3_ with a lower oxidation state than that of LaCoO_3_ enables stabilization of O_2_ (ads), while Mn cationic sites mainly act as promoters by increasing the adsorption strength of CO (ads) and NO (ads), as well as altering the oxidation state of the Co sites and facilitating oxygen vacancy formation and vacancy refilling with oxygen.

Consequently, currently reported findings establish a strong correlation between the structural properties of the LCM catalysts composed of different types of B-site cations with different loadings and their corresponding enhanced catalytic performances in oxidation reactions, demonstrating that the catalytic behavior of these perovskites can be optimized for specific reactions by fine-tuning their electronic features at the nanometer scale.

## Supplementary Material



## Notes

Precursor amounts for synthesized perovskites; configuration of the catalytic test reactor; NO and CO oxidation reaction steps; reaction conditions for short-term catalytic durability tests; calculation of NO oxidation equilibrium conversion values at various temperatures; TEM images of LaCo_0.7_Mn_0.3_O_3_; BET and ICP-MS results for the synthesized perovskites; XPS relative surface elemental composition results; details of *ex-situ* and *in situ* XANES measurements; and relative amounts of desorption products obtained from NO_*x*_-TPD experiments (PDF)
