# Supplementary material for: Cooperative Catalytic Role of Co and Mn Sites on LaCo x Mn1–x O3 Perovskite Nanoparticles in CO and NO Oxidation
Source: ACS Appl Nano Mater. 2025 Aug 18;8(34):16779–91. doi: 10.1021/acsanm.5c02876 (PMC12400280; doi:10.1021/acsanm.5c02876)
Supplement: Supplementary file 1 [file an5c02876_si_001.pdf]

SUPPORTING INFORMATION

Cooperative Catalytic Role of Co and Mn Sites on $LaCo_xMn_{1-x}O_3$ Perovskites Nanoparticles in CO and NO Oxidation

Kerem Emre Ercan,[†, ¥] *Mustafa Karatok,*[‡] *Zafer Say,*[†, ¥] *Merve Kurt,*[†] *Abel Tetteh Sika-Nartey,*[†] *and Emrah Ozensoy* [*, †, ¥]

[†]Department of Chemistry, Bilkent University, 06800, Ankara, Turkiye;
[¥]Roketsan Inc., 06780 Elmadag, Ankara, Turkiye;
‡Department of Nanotechnology and Nanomedicine, Hacettepe University, 06800, Ankara, Turkiye;
[¥]Department of Materials Science and Nanotechnology Engineering TOBB University of Economics and Technology, 06510, Ankara, Turkiye;
[¥]UNAM-National Nanotechnology Center, Bilkent University, 06800, Ankara, Turkiye.

*Corresponding Author: Emrah Ozensoy; e-mail: ozensoy@fen.bilkent.edu.tr

List of Contents

S1. Catalyst Preparation……………………………………………………..…………….....3

S2. Catalytic Performance Experiments………………………………...…………..…..3

S2.1. Reactor Configuration ………………………………………………...………..……3

S2.2. NO Oxidation Reaction Steps …..……………………………………………………4

S2.3. CO Oxidation Reaction Steps …..……………………………………………………5

S2.4. Short Term Durability Tests …..…………………………………...………...…7

S2.5.Calculation of NO Oxidation Equilibrium Conversion Values ……………………......……7

S3. Structural Characterization of the Catalysts ………..………………………………...……..…8

S3.1. TEM Images of $LaCo_{0.7}Mn_{0.3}O_3$ Catalyst …………………………..………………….…8

S3.2. BET Measurements for the Synthesized Perovskite Samples …….………..……….……...9

S3.3. ICP-MS Measurements…………………………………………….……………..….……….9

S3.4. Surface Atomic Compositional Analysis via XPS ………..……………………..…..…10

S3.5. Ex-situ XANES Experiments ……………………………………………………......….11

S3.6. In-situ XANES Experiments ………………………………………………..……......13

S3.7. NO_x TPD Experiments …………………………………………………...……..…..…14

S4. References …………………………………………………….……………..……..…14

S1. Catalyst Preparation

Table S1. Precursor amounts for the synthesized perovskites.

$LaCo_xMn_{1-x}O_3$	$La(NO_3)_3.6H_2O$ (g)	$Mn(NO_3)_2.4H_2O$ (g)	$Co(NO_3)_2.6H_2O$ (g)	Citric Acid (g)	De-ionized Water (ml)
x = 0	1.791	1.038	-	1.912	82.7
x = 0.2	1785	0.828	0.240	1.902	82.4
x = 0.4	1.779	0.619	0.478	1.899	82.2
x = 0.6	1.773	0.411	0.715	1.893	82.0
x = 0.7	1.770	0.308	0.833	1.890	81.8
x = 0.8	1.767	0.205	0.950	1.887	81.6
x = 1.0	1.761	-	1.184	1.881	81.3

S2. Catalytic Performance Experiments

S2.1 Reactor configuration

The reactor setup (**Figure S1**) was equipped with 9 different Mass Flow Controllers (MFC) (Hastings® HFC-202), thermocouples (TC) for temperature sensing, Proportion Integration Derivation (PID) controllers for controlling the reactor temperature as well as the temperature of the gas lines, Pfeiffer Hi-Cube 80 Turbomolecular Pumping Station, Bayard-Alpert type ionization gauge, and custom-design electronics for experimental control. A quartz tube (47 cm long with a 1 cm outer diameter, 0.1 cm wall thickness and a node to place powder catalysts) was used as the catalytic reactor.

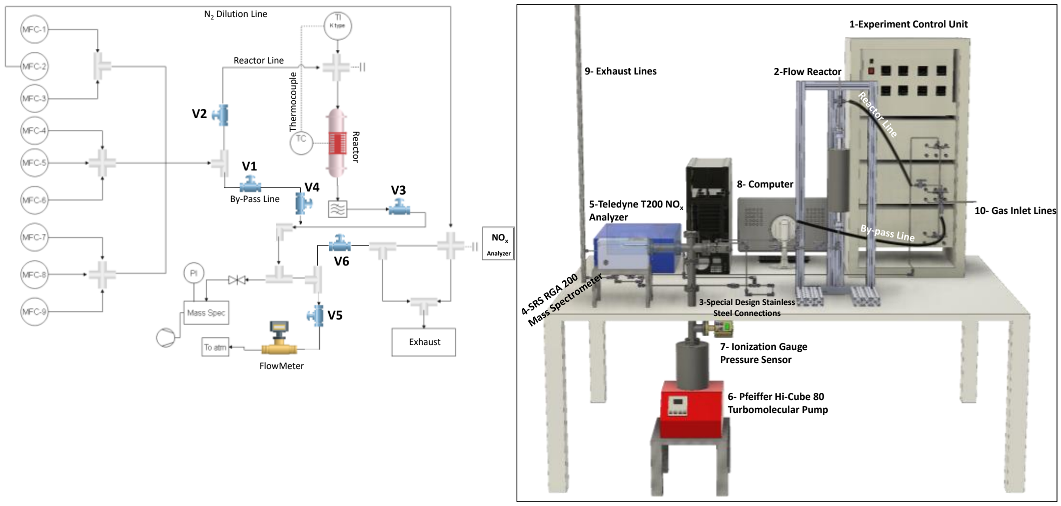

Figure S1. Custom-design computer-controlled flow mode catalytic performance test reactor used in the current catalytic performance and long-term catalytic stability tests.

S2.2. NO oxidation reaction steps

The following procedure outlines the sequential steps for annealing and gas treatment of the sample to evaluate its catalytic performance in NO oxidation (**Figure S2**).

Step 1: Anneal in 4 % H_2/Ar within 320-600 K with a 6 K/min heating ramp, total flow rate of 500 ml (STP) min^{-1}. Then, soak at 600 K with the same gas feed for 1 h.

Step 2: Switch the gas feed to 20 % O_2/Ar at 600 K with a total flow rate of 500 ml (STP) min^{-1} and soak for 1 h. Then cool the sample in this gas feed to 320 K.

Step 3: Switch the gas feed to 700 ppm NO and 8 % O_2 in Ar at 320 K with a total flow rate of 500 ml (STP) min^{-1} and anneal with a heating ramp of 6 K/min to 723 K. Soak at 723 K for 30 min and cool to 320 K in the same gas feed.

Step 4: Switch the gas feed to 4 % H_2/Ar at 320 K and anneal to 973 K with a 6 K/min heating ramp, total flow rate of 500 ml (STP) min^{-1}. Then, soak at 973 K with the same gas feed for 1 h and cool to 320 K in the same gas feed.

Step 5: Switch the gas feed to 700 ppm NO and 8 % O_2 in Ar at 320 K with a total flow rate of 500 ml (STP) min^{-1} and anneal with a heating ramp of 4 K/min to 723 K. Soak at 723 K for 30 min and cool to 320 K in the same gas feed. Performance data **Figure 2a** of the main text is recorded during this latter stage. For the NO oxidation catalytic performance tests, gas hourly space velocity (GHSV) and volume hourly space velocity (VHSV) values were calculated as 50,000 h^{-1}/1,000,000 cm^3(STP).$gcat^{-1}$.h^{-1}/1,073,000 Ncm^3.$gcat^{-1}$.h^{-1}, respectively.

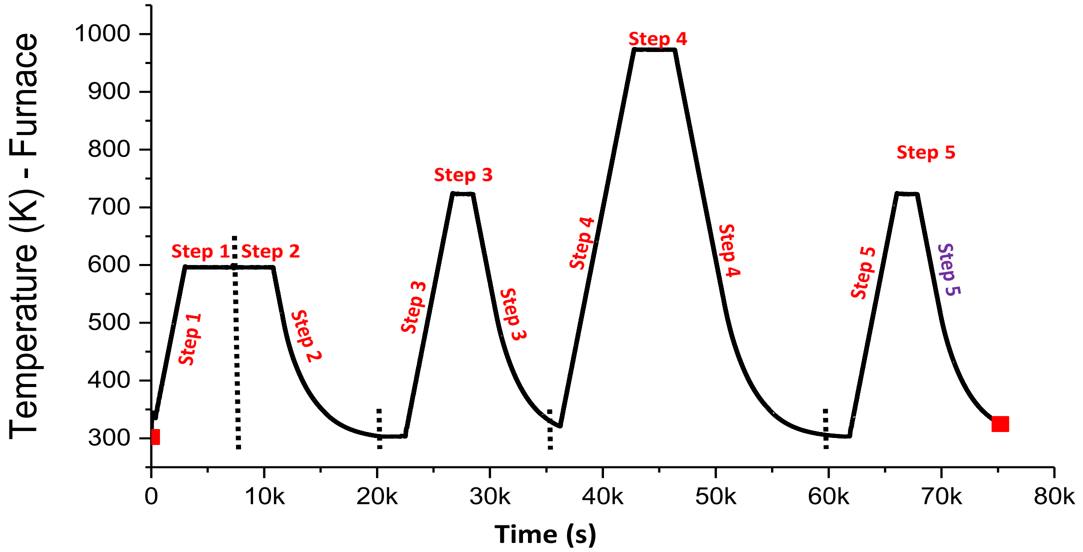

Figure S2. Description of the particular steps used in the catalytic NO oxidation performance tests.

The NO conversion % is defined as follows, where $(NO)_{in}$ and $(NO)_{out}$ correspond to the reactor inlet and reactor outlet NO concentrations, respectively:

$$NO\ \ conversion\ \% = \left|\frac{(NO)_{in} - (NO)_{out}}{(NO)_{in}}\right|\ X\ 100$$

Specific Reaction Rate (SRR) is calculated as:

$$n_{(\mu mol)} = \frac{P_{(atm)} \cdot V_{(L)}}{R\ .\ T_{(K)}}$$

$$SRR(\mu mol\ NO.gcat^{-1}.s^{-1}) = n_{(\mu mol)}\ .m_{(g)}^{-1}\ .t_{(s)}^{-1}\ .c_{(NO)}\ .10^{-6}.\ \frac{a_{(NO\ \%)}}{100}$$

Where:

$P_{(atm)} = Standard\ Pressure =\ 1\ atm$

$V_{(L)}$

$= Total\ volume\ of\ reactants\ and\ the\ Ar(g)\ balance\ gas\ passing\ through\ the\ reactor\ in\ 1\ h$

$T_{(K)} = Standard\ Temperature\ = 273\ K$

$R = Universal\ Gas\ constant$

$n_{(\mu mol)} = Total\ number\ of\ moles\ of\ NO\ passing\ through\ the\ reactor\ in\ 1\ h$

$m_{(g)} =\ Mass\ of\ the\ catalyst\ in\ grams$

$t_{(s)} =\ Reaction\ duration\ (i.e., 1\ h\ )$

$c_{(NO)} =\ Concentration\ of\ NO\ in\ ppm$

$$a_{(NO\ \%)} = NO\ conversion\ \%$$

S2.3. CO oxidation reaction steps

The following procedure outlines the sequential steps for annealing and gas treatment of the sample to evaluate its catalytic performance in CO oxidation (**Figure S3**):

Step 1: Anneal in 4 % H_2/Ar within 320-600 K with a 6 K/min heating ramp, total flow rate of 500 ml (STP) min^{-1}. Then, soak at 600 K with the same gas feed for 1h.

S*tep 2:* Switch the gas feed to 20 % O_2/Ar at 600 K with a total flow rate of 500 ml (STP) min^{-1} and soak for 1h. Then cool the sample in this gas feed to 320 K.

Step 3: Switch the gas feed to 1.6 % CO and 20 % O_2 in Ar at 320 K with a total flow rate of 500 ml (STP) min^{-1} and anneal with a heating ramp of 4 K/min to 773 K. Soak at 773 K for 30 min and cool to 320 K in the same gas feed.

Step 4: Switch the gas feed to 4 % H_2/Ar at 320 K and anneal to 973 K with a 6 K/min heating ramp, total flow rate of 500 ml (STP) min^{-1}. Then, soak at 973 K with the same gas feed for 1 h and cool to 320 K in the same gas feed.

Step 5: Switch the gas feed to 1.6 % CO and 20 % O_2 in Ar at 320 K with a total flow rate of 500 ml (STP) min^{-1} and anneal with a heating ramp of 4 K/min to 773 K. Performance data given in **Figure 2b** of the main text is recorded during this stage. During the CO oxidation catalytic performance tests, gas hourly space velocity (GHSV) and volume hourly space velocity (VHSV) values were calculated as 100,000 h^{-1}/750,000 cm^3 (STP).g^{-1}.h^{-1}/ 805,000 Ncm^3.g^{-1}.h^{-1}, respectively.

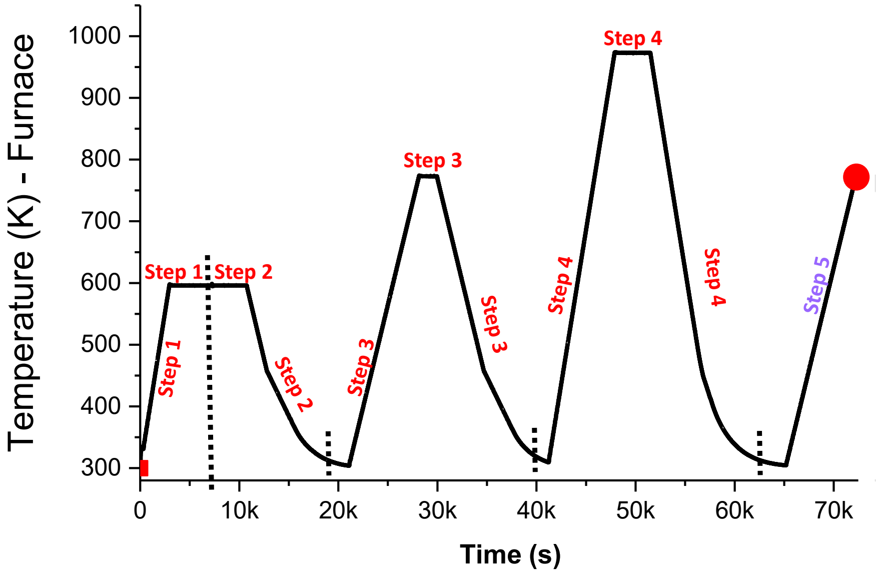

Figure S3. Description of the particular steps used in the catalytic CO oxidation performance tests.

Additionally, T200 Teledyne Chemiluminescent NO_x analyzer that was utilized in the current catalytic NO oxidation experiments for reactant/product NO/NO_2 detection precludes the addition of water to the feed gas mixture as it leads to interferences in NO/NO_2 detection, depletion of ozone used in the NO_x detection method and formation of highly corrosive HNO_3 in the analyzer.

The CO conversion % is defined as follows, where $(CO)_{in}$ and $(CO)_{out}$ correspond to the reactor inlet and reactor outlet CO concentrations, respectively:

$$O\ Conversion\ \% = \left|\frac{(CO)_{in} - (CO)_{out}}{(CO)_{in}}\right|\ X\ 100$$

Specific Reaction Rate (SRR) is calculated as:

$$n_{(\mu mol)} = \frac{P_{(atm)} \, . \, V_{(L)}}{R \, . \, T_{(K)}}$$

$$SRR(\mu mol\ CO.gcat^{-1}.s^{-1}) = n_{(\mu mol)} \, . \, m_{(g)}^{-1} \, . \, t_{(s)}^{-1} \, . \, c_{(CO)} \, . \, 10^{-2} . \frac{a_{(CO\,\%)}}{100}$$

where:

$P_{(atm)} = Standard\ pressure = 1\ atm$
$V_{(L)}$
$= Total\ volume\ of\ reactants\ and\ the\ Ar(g)\ balance\ gas\ passing\ through\ the\ reactor\ in\ 1\ h$
$T_{(K)} = Standard\ temperature = 273\ K$
$R = Universal\ gas\ constant$
$n_{(\mu mol)} = Total\ number\ of\ moles\ of\ CO\ passing\ through\ the\ reactor\ in\ 1\ h$
$m_{(g)} = Mass\ of\ the\ catalyst\ in\ grams$
$t_{(s)} = Reaction\ duration\ (i.e., 1\ h = 3600\ s)$
$c_{(CO)} = \%\ CO\ concentration$
$a_{(CO\,\%)} = CO\ conversion\ \%$

Our preliminary results (data not shown) indicate that catalytic steady state could be reached using the particular pretreatment conditions given in **Figure S2-S3**. Accordingly, currently chosen harsh pretreatment conditions also ensure reliable de-greening of the investigated catalysts.

S2.4. Short-term Catalytic Durability Tests

In the short-term catalytic stability experiments, each catalyst was initially pretreated and de-greened by using the same procedure for NO/CO oxidation given in **Figure S2-S3**. To obtain isothermal conditions in the catalyst bed, 50 mg of catalyst was mixed with 960 mg α-Al_2O_3 diluent for the short-term catalytic stability CO oxidation experiment. The active material and diluent amount for the short-term NO oxidation stability experiments were kept same as it was mentioned in the main text. The short-term stability experiments were carried out isothermally at 600 K for NO oxidation reaction and at 410 K for CO oxidation reaction. Both stability tests lasted 28 h. Temperatures used in the short-term stability tests were intentionally chosen so that these values are below the corresponding temperatures for equilibrium conversion.

S2.5. Calculation of NO Oxidation Equilibrium Conversion Values at Various Temperatures

Chemical equilibrium compositions for the reaction system NO + O_2 in a gas mixture initially containing 500 ppm NO, 8% O_2, and Ar gas (balance Ar) were computed using ThermoSolver, a Gibbs energy minimization software (Barnes, C. S.; Koretsky, M. D. ThermoSolver Software; John Wiley and Sons: Hoboken, NJ, 2004. Accompanies the textbook Engineering and Chemical Thermodynamics, 2nd ed., by M. D. Koretsky). The software solves the equilibrium composition by minimizing the total Gibbs free energy subject to mass balance constraints using the Newton–Raphson method, with a fallback to the Simplex method for better convergence.

In this system, the total mole fraction inputs correspond to:

NO = 500 ppm = 5×10^{-4} mol/mol

O_2 = 0.08 mol/mol
NO_2 = equilibrium species (varies based on system conditions)
Ar = balance (remaining fraction of the mixture)

ThermoSolver minimizes the following equation for each species i:

$$\Delta_{g_i}{}^{f} + RTln\left(\frac{f_i}{f_i{}^{o}}\right) + \sum_{j=1}^{i} \lambda_j \beta_{ij} = 0$$

where the fugacity is approximated as $f_i = y_i P$ for gases, with y_i being the mole fraction and P being the system pressure (assumed to be 1 bar unless otherwise noted). The mole amounts n_i are parameterized as:

$$n_i = exp(x_i)$$

to ensure positivity during optimization. The Lagrange multipliers λ_j enforce elemental balance, while the objective function (total G) is iteratively minimized. This method enables the prediction of equilibrium species such as NO_2, depending on the system temperature and pressure.

S3. Structural Characterization of the Catalysts

S3.1. TEM Images of $LaCo_{0.7}Mn_{0.3}O_3$ Catalyst

TEM images of the $LaCo_{0.7}Mn_{0.3}$ catalyst are given in **Figure S4**. It is apparent that the particles are similar in size, averaging approximately 30-40 nm. Atomically well-ordered and crystalline nature of these perovskites can be seen in high resolution images in **Figure S4b-S4d**.

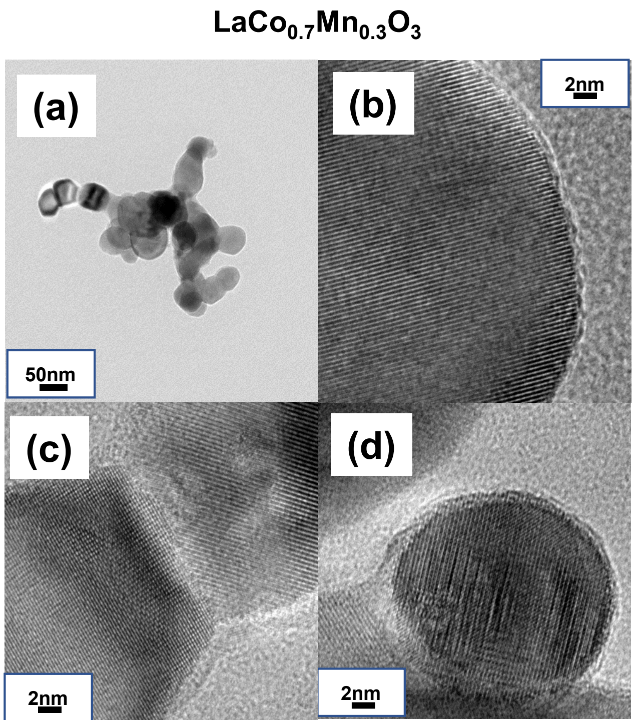

Figure S4. (a-d) TEM bright field images of the $LaCo_{0.7}Mn_{0.3}O_3$ catalyst.

S3.2. BET Measurements for the Synthesized Perovskite Samples

BET specific surface area measurements for the currently synthesized $LaCo_xMn_{1-x}O_3$ perovskites (x = 0, 0.2, 0.4, 0.6, 0.7, 0.8, 1.0) are presented in **Figure S5**. $LaMnO_3$ reveals a relatively higher SSA (21 m^2/g) than that of $LaCoO_3$ (8 m^2/g), while SSA of $LaCo_xMn_{1-x}O_3$ catalysts are located in between these two extremes. SSA values of these $LaMnO_3$ and $LaCoO_3$ samples are consistent with the former studies in the literature.[1-3] It is apparent that the increasing Mn loading in the $LaCo_xMn_{1-x}O_3$ structure typically tends to increase the SSA.

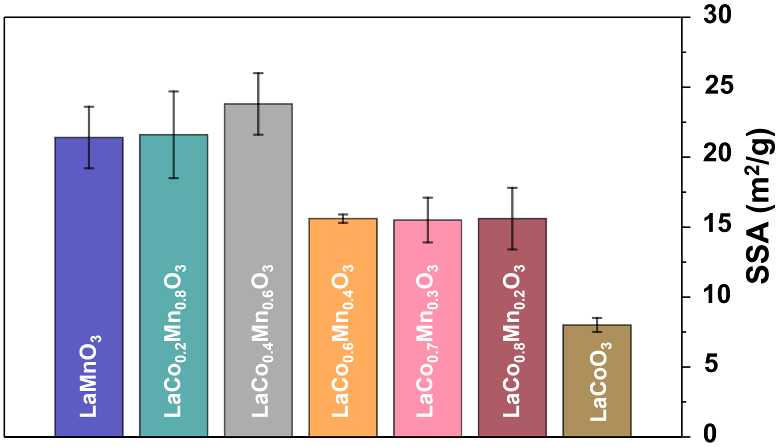

Figure S5. BET specific surface area (SSA) values of the currently synthesized $LaMnO_3$, $LaCoO_3$ simple perovskites and $LaCo_xMn_{1-x}O_3$ ($0 \leq x \leq 1.0$).

S3.3. ICP-MS Measurements

Table S2. Variance (s^2) and standard deviation (s) values for the elemental composition data obtained from ICP-MS measurements (i.e., 5 independent measurements per sample).

Perovskites with Nominal Composition ($La_xCo_yMn_zO_3$)	*Variance (s^2) for x (La)*	*Standard Deviation (s) for x (La)*	*Variance (s^2) for y (Co)*	*Standard Deviation (s) for y (Co)*	*Variance (s^2) for z (Mn)*	*Standard Deviation (s) for z (Mn)*
**$LaCoO_3$**	2.0×10^{-4}	0.014	0.35×10^{-4}	0.006	N/A	N/A
**$LaCo_{0.8}Mn_{0.2}O_3$**	1.3×10^{-4}	0.011	0.66×10^{-4}	0.008	0.2×10^{-5}	0.001
**$LaCo_{0.7}Mn_{0.3}O_3$**	0.5×10^{-4}	0.007	0.39×10^{-4}	0.006	0.8×10^{-5}	0.003
**$LaMnO_3$**	2.1×10^{-4}	0.014	N/A	N/A	3.6×10^{-5}	0.006

S3.4. Surface Atomic Compositional Analysis via XPS

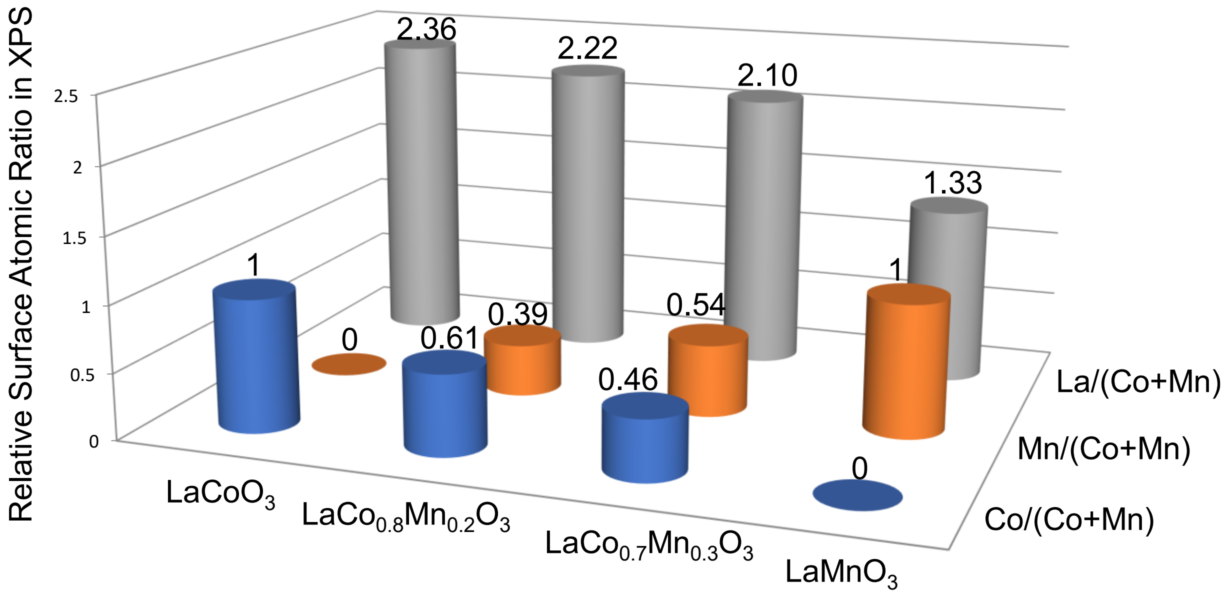

Figure S6. Relative surface atomic compositional analysis via XPS for the $LaMnO_3$, $LaCoO_3$ simple perovskites and the best performing $LaCo_xMn_{1-x}O_3$ catalysts (i.e., $LaCo_{0.7}Mn_{0.3}O_3$, $LaCo_{0.8}Mn_{0.2}O_3$).

S3.5. *Ex-situ* XANES Experiments

Table S3. Variance (s^2) and standard deviation (s) calculation results for bulk oxidation state (q_{Co}, q_{Mn}) determination obtained via XANES experiments (i.e., 3 *ex-Situ* XANES measurements per sample).

Perovskites with Nominal Composition ($La_xCo_yMn_zO_3$)	*Variance (s^2) for q_{Co}*	*Standard Deviation (s) for q_{Co}*	*Variance (s^2) for q_{Mn}*	*Standard Deviation (s) for q_{Mn}*
**$LaCoO_3$**	1.6 x 10^{-4}	0.012	N/A	N/A
**$LaCo_{0.8}Mn_{0.2}O_3$**	1.6 x 10^{-4}	0.013	0.3 x 10^{-4}	0.006
**$LaCo_{0.7}Mn_{0.3}O_3$**	0.3 x 10^{-4}	0.005	0.3 x 10^{-4}	0.005
**$LaMnO_3$**	N/A	N/A	8.0 x 10^{-4}	0.003

Figures 3c-3d in the main text show the *ex-situ* XANES Mn K-edge and Co K-edge data for tested perovskites, and corresponding benchmark metal oxide compounds revealing the gradual shift of Mn and Co bulk oxidation states as a function of varying synthetic parameters. Shifts of Mn and Co K edges can be useful guides to estimate and monitor the bulk oxidation states of the synthesized perovskites.[4-7] In the bulk oxidation state analysis, CoO, Co^{3+}, MnO, Mn_2O_3, and MnO_2 samples with well-known oxidation states were used as reference materials. Using these reference XANES data, the linear calibration curve (Figure 3e) was obtained which depicts the edge energy shift of the B-site cation with respect to its metallic state versus the average bulk oxidation state of the B-site cation.

Figure S7 presents the La L-III edge *ex-situ* XANES measurements for La_2O_3, $LaCoO_3$, $LaCo_{0.8}Mn_{0.2}O_3$, $LaCo_{0.7}Mn_{0.3}O_3$, $LaMnO_3$ under ambient and reducing (5 % H_2/He mixture at 773 K) conditions. Similar La L-III edge energies for perovskites and La_2O_3 confirm that the oxidation state of La cations in the perovskites is +3.[8] Note that $LaCo_{0.8}Mn_{0.2}O_3$ XANES spectrum was acquired in transmission mode whereas all other spectra were recorded in fluorescence mode in order to improve the signal to noise ratio. Thus, the relatively different white-line intensity (WLI) of $LaCo_{0.8}Mn_{0.2}O_3$ when compared to other perovskites is due to the difference in detection technique. WLI differences between reference lanthanum oxide and perovskite samples might be associated with the filling level of the La 5d orbitals. Former studies in the literature reported that WLI can increase or decrease for a fixed La oxidation state of +3.[8, 9]

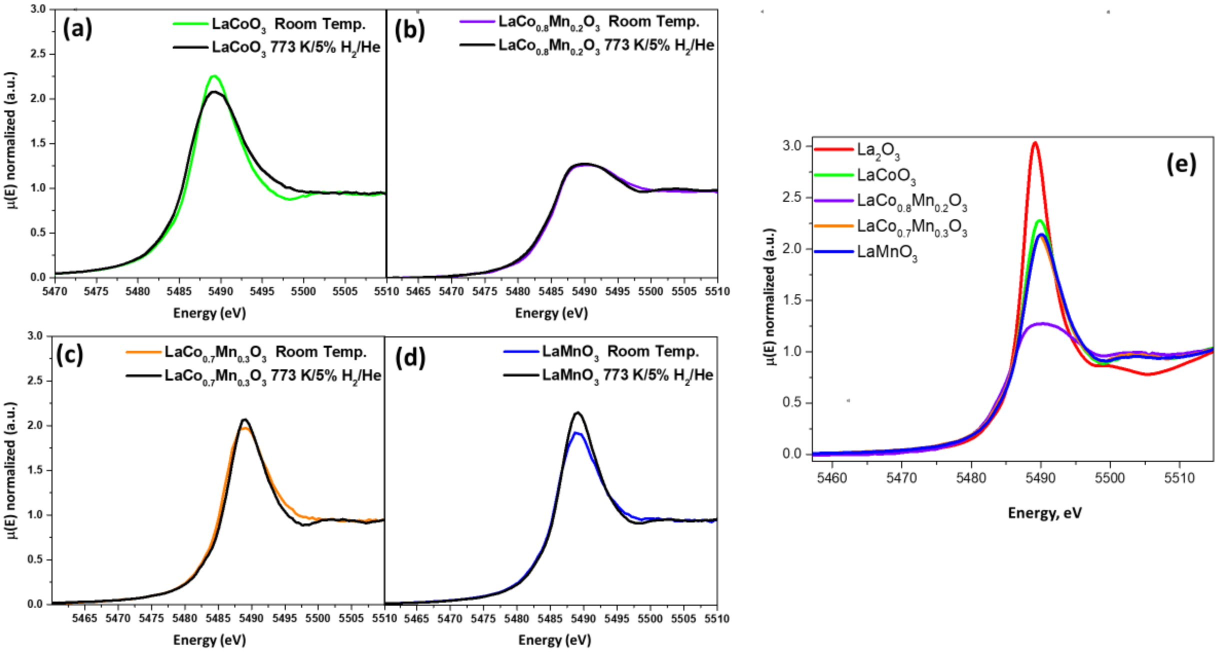

Figure S7. Comparison of La edge for a) $LaCoO_3$, b) $LaCo_{0.8}Mn_{0.2}O_3$, c) $LaCo_{0.7}Mn_{0.3}O_3$, and, d) $LaMnO_3$ under ambient and reducing (in 5 % H_2/He mixture at 773 K) conditions. e) Ex-situ XANES La L-III edge data for La_2O_3, $LaCoO_3$, $LaCo_{0.8}Mn_{0.2}O_3$, $LaCo_{0.7}Mn_{0.3}O_3$, $LaMnO_3$.

S3.6. *In-situ* XANES Experiments

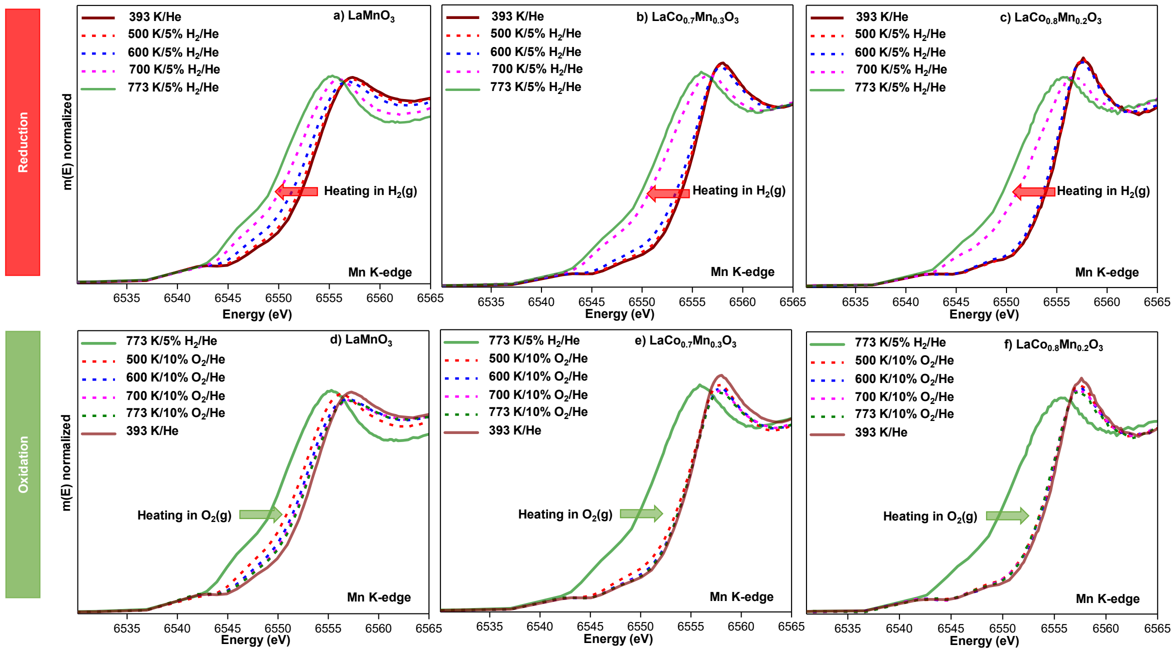

Figure S8. In-situ XANES Mn-K edge data where bulk oxidation states of B-site cations of $LaMnO_3$ (a,d), $LaCo_{0.7}Mn_{0.3}O_3$ (b,e), and $LaCo_{0.8}Mn_{0.2}O_3$ (c,f) were monitored in real time under subsequent H_2(g) and O_2(g) flow at various temperatures.

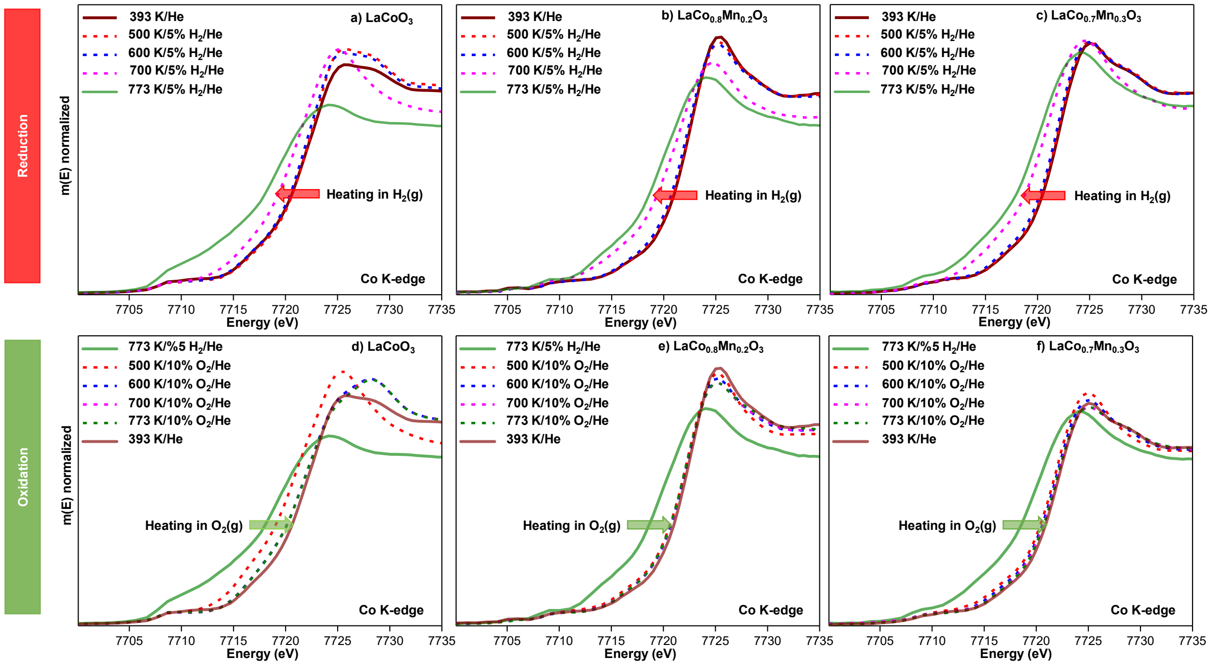

Figure S9. In-situ XANES Co-K edge data where bulk oxidation states of B-site cations of $LaCoO_3$ (a,d), $LaCo_{0.8}Mn_{0.2}O_3$ (b,e), and $LaCo_{0.7}Mn_{0.3}O_3$ (c,f) were monitored in real time under subsequent H_2 (g) and O_2 (g) flow at various temperatures.

S3.7. NO_x TPD Experiments

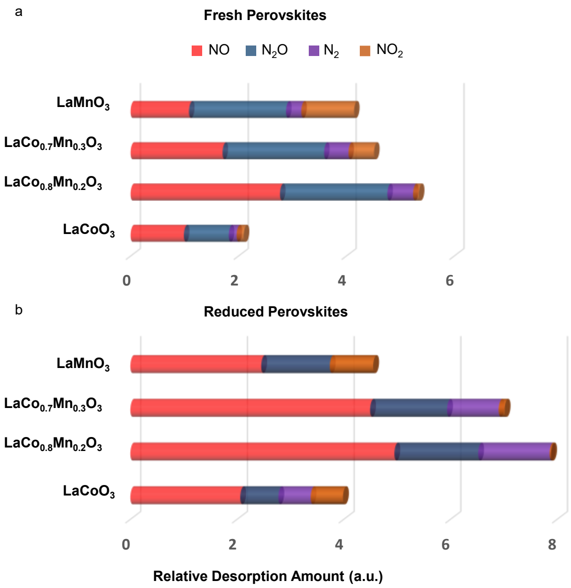

Figure S10. NO_x-TPD results upon NO_2(g) adsorption at 323 K on a) fresh and b) pre-reduced forms of $LaMnO_3$, $LaCoO_3$, $LaCo_{0.7}Mn_{0.3}O_3$, and $LaCo_{0.8}Mn_{0.2}O_3$ with H_2 at 623 K. Particular relative quantity of each desorbing gas species was derived from the experimental TPD data using the corresponding mass spectroscopic fragmentation factors of the desorbing gases.

S4. References

(1) Say, Z.; Dogac, M.; Vovk, E. I.; Kalay, Y. E.; Kim, C. H.; Li, W.; Ozensoy, E. Palladium doped perovskite-based NO oxidation catalysts: The role of Pd and B-sites for NOx adsorption behavior via in-situ spectroscopy. Appl. Catal., B **2014**, 154–155, 51–61. https://doi.org/10.1016/j.apcatb.2014.01.038

(2) Civera, A.; Pavese, M.; Saracco, G.; Specchia, V. Combustion synthesis of perovskite-type catalysts for natural gas combustion. Catal. Today **2003**, 83 (1–4), 199–211. https://doi.org/10.1016/S0920-5861(03)00220-7

(3) Ziaei-Azad, H.; Khodadadi, A.; Esmaeilnejad-Ahranjani, P.; Mortazavi, Y. Effects of Pd on enhancement of oxidation activity of $LaBO_3$ (B=Mn, Fe, Co, and Ni) perovskite catalysts for pollution abatement from natural gas fueled vehicles. Appl. Catal., B **2011**, 102 (1–2), 62–70. https://doi.org/10.1016/j.apcatb.2010.12.016

(4) Sikora, M.; Kapusta, C.; Knížek, K.; Jirák, Z.; Autret, C.; Borowiec, M.; Oates, C. J.; Procházka, V.; Rybicki, D.; Zajac, D. X-ray absorption near-edge spectroscopy study of Mn and Co valence states in $LaMn_{1-x}Co_xO_3$ (x= 0-1). *Physical Review B* **2006**, *73* (9), 094426. https://doi.org/10.1103/PhysRevB.73.094426

(5) Croft, M.; Sills, D.; Greenblatt, M.; Lee, C.; Cheong, S. W.; Ramanujachary, K. V.; Tran, D. Systematic Mn d-configuration change in the $La_{1-x}Ca_xMnO_3$ system: A Mn K-edge XAS study. *Physical Review B* **1997**, *55* (14), 8726-8732. https://doi.org/10.1103/PhysRevB.55.8726

(6) Sánchez, M. C.; García, J.; Blasco, J.; Subías, G.; Perez-Cacho, J. Local electronic and geometrical structure of $LaNi_{1-x}Mn_xO_{3+\delta}$ perovskites determined by x-ray-absorption spectroscopy. *Physical Review B* **2002**, *65* (14), 144409. https://doi.org/10.1103/PhysRevB.65.144409

(7) De Vries, A.; Hozoi, L.; Broer, R. Origin of the chemical shift in X-ray absorption near-edge spectroscopy at the Mn K-edge in manganese oxide compounds. *International journal of quantum chemistry* **2003**, *91* (1), 57-61. https://doi.org/10.1002/qua.10370

(8) Lawley, C.; Nachtegaal, M.; Stahn, J.; Roddatis, V.; Döbeli, M.; Schmidt, T. J.; Pergolesi, D.; Lippert, T., Examining the surface evolution of $LaTiO_xN_y$ an oxynitride solar water splitting photocatalyst. *Nat. Commun.* **2020,** *11* (1), 1728-1728. https://doi.org/10.1038/s41467-020-15519-y

(9) Ishimatsu, N.; Sasada, R.; Maruyama, H.; Ichikawa, T.; Miyaoka, H.; Kimura, T.; Tsubota, M.; Kojima, Y.; Tsumuraya, T.; Oguchi, T.; Kawamura, N.; Machida, A., Effect of hydrogenation on the electronic state of metallic La hydrides probed by X-ray absorption spectroscopy at the La L-edges. *J. Phys. Conf. Ser.* **2009,** *190*, 012070. https://doi.org/10.1088/1742-6596/190/1/012070
